# Decoding the olfactory map through targeted transcriptomics links murine olfactory receptors to glomeruli

**DOI:** 10.1038/s41467-022-32267-3

**Published:** 2022-09-01

**Authors:** Kevin W. Zhu, Shawn D. Burton, Maira H. Nagai, Justin D. Silverman, Claire A. de March, Matt Wachowiak, Hiroaki Matsunami

**Affiliations:** 1 Department of Molecular Genetics and Microbiology, Duke University School of Medicine, Durham, NC 27710 USA; 2 Department of Biological Sciences, Lehigh University, Bethlehem, PA 18015 USA; 3 Department of Neurobiology, University of Utah School of Medicine, Salt Lake City, UT 84112 USA; 4 College of Information Science and Technology, Pennsylvania State University, University Park, PA 16802 USA; 5 Department of Statistics, Pennsylvania State University, University Park, PA 16802 USA; 6 Department of Medicine, Pennsylvania State University, Hershey, PA 17033 USA; 7 Institute for Computational and Data Science, Pennsylvania State University, University Park, PA 16802 USA; 8 Department of Neurobiology, Duke University School of Medicine, Durham, NC 27710 USA; 9 Duke Institute for Brain Sciences, Duke University, Durham, NC 27710 USA

**Keywords:** Olfactory bulb, Gene expression analysis

## Abstract

Sensory processing in olfactory systems is organized across olfactory bulb glomeruli, wherein axons of peripheral sensory neurons expressing the same olfactory receptor co-terminate to transmit receptor-specific activity to central neurons. Understanding how receptors map to glomeruli is therefore critical to understanding olfaction. High-throughput spatial transcriptomics is a rapidly advancing field, but low-abundance olfactory receptor expression within glomeruli has previously precluded high-throughput mapping of receptors to glomeruli in the mouse. Here we combined sequential sectioning along the anteroposterior, dorsoventral, and mediolateral axes with target capture enrichment sequencing to overcome low-abundance target expression. This strategy allowed us to spatially map 86% of olfactory receptors across the olfactory bulb and uncover a relationship between OR sequence and glomerular position.

## Abstract (web summary)

Targeted spatial transcriptomics mapped olfactory receptor mRNAs to sections of the murine olfactory bulb to generate an interactive, statistical, 3D model of glomeruli locations and identify an ultra-sensitive receptor-odorant relationship.

## Introduction

The organization of axonal projections from olfactory sensory neurons (OSNs) in the olfactory epithelium (OE) to glomeruli on the olfactory bulb (OB) forms the olfactory map^1–4^. In the mouse, each canonical OSN expresses a single olfactory receptor (OR) or trace amine-associated receptor (TAAR) allele from a repertoire of over 1000 OR and TAAR genes. Insights into the organization of the olfactory map were first obtained using in situ hybridization, where OR transcript probes indicated the convergence of OSN axons into discrete structures called glomeruli on the OB surface, which range from 50 to 120 μm in diameter^3,5,6^. Later, this organization was more clearly visualized through the use of gene-targeted mouse lines, which demonstrated that glomeruli are formed from the axonal convergence of OSNs expressing the same OR gene^7^. Together these studies established the convergence of homotypic OSN axons to stereotyped glomeruli whose positional variability ranges from 75 to 270 μm depending on OR identity^5,8^. Because each glomerulus represents a single OR and a single odorant can bind multiple ORs, odor signals detected in the OE are transformed into a map of glomerular activity on the OB^9–13^.

To date, glomerular positions for less than 5% of mouse ORs are available, and further progress has been stymied due to the low-throughput, laborious, and time-consuming aspects of currently available methodologies for mapping each OR in the expansive murine repertoire^7,8,13–29^. Furthermore, the ability to compare locations between multiple glomeruli is limited among these studies due to the lack of reference landmarks on the OB and differences between methodologies. A more efficient approach for mapping OR axon projections to OB glomeruli would serve to generate a more comprehensive and informative map that would serve as a foundation for further functional studies of odor coding and processing. In this study, we demonstrated that target capture enrichment on sequential samples from the OB enables detection of low-abundance OR and TAAR mRNA in the axon termini of OSNs. Using this approach, we mapped 86% of the 1118 ORs and TAARs along the anteroposterior, mediolateral, and dorsoventral axes and combined these data to generate a three-dimensional model of glomerular positions with a precision of 141 μm. We examined the relationship between OR sequence and OB position, identified the set of ORs and TAARs expressed within dorsal glomeruli accessible to functional imaging, and generated gene-targeted mouse lines for two dorsal glomerular ORs amenable to functional characterization in vivo.

## Results

### Targeted capture consistently enriches OR transcripts

**Fig. 1:**
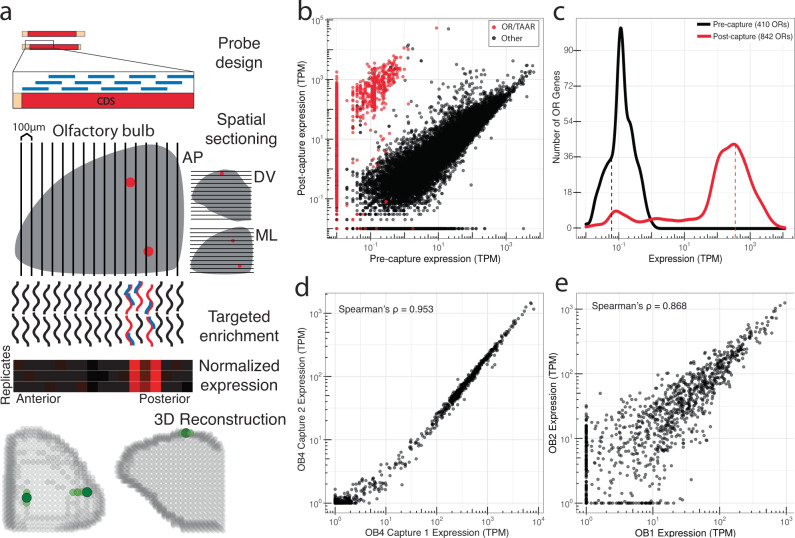
Target capture sequencing consistently enriches OR transcripts. **a** Methodological overview for targeted enrichment of OR and TAAR transcripts from OB sections. Briefly, RNA is extracted from OB tissue and used for cDNA synthesis and library preparation. Biotinylated capture probes are designed against coding sequences (CDS) of interest, enabling enrichment of target genes following streptavidin binding and washing steps. RNA-Seq of enriched libraries allows for high-throughput positional analyses when combined with systematic tissue sectioning in both individual dimensions and in 3D after reconstruction. **b** Pre- and post-capture normalized abundances (transcripts per million; TPM) of intact OR and TAAR genes (red) and other intact genes (black) from a whole OB. Source data provided as a Source Data file. **c** Frequency distribution of OR gene abundances pre- and post-capture from a whole OB. **d** Technical replicates demonstrating OR and TAAR gene abundances from independent capture enrichments of the same whole-OB RNA. **e** Biological replicates demonstrating OR and TAAR gene abundances from capture enrichment of whole-OB RNA samples from different animals.

Previous studies have detected low levels of OR mRNA in OSN axon terminals, identifying the glomerular positions for specific ORs within histological sections^5,23,27,30^. To quantify OR and TAAR transcripts in the OB we first performed conventional RNA-Seq on whole-OB tissue from a mouse at postnatal day 21, an age when olfactory glomeruli are fully developed and finalized in their stereotyped positions^31,32^. Quantification of 25.7 million reads identified 410/1118 (36.7%) intact ORs at an average abundance of 0.06 transcripts per million (TPM) (median OR TPM = 0, 6/15 (40%) TAARs with TPM above 0, mean TAAR TPM = 0.077, median TAAR TPM = 0) (Fig. 1c), confirming the low abundance of OR mRNA in OSN axon terminals^30,33^.

To enrich sampling for OR transcripts, we designed a target capture array against chemosensory receptor gene families primarily targeting OR and TAAR mRNA^34^. We applied this target capture array to the previously sequenced OB library and identified 842 of the 1118 ORs (75.3%) and 10 of the 15 TAARs (66.7%) (Fig. 1a–c). Following targeted capture, these OR mRNAs were present in a set of 27.7 million reads at an average abundance of 360.27 TPM (median = 106.74 TPM, mean TAAR TPM = 871.66, median TAAR TPM = 64.62) resulting in a mean fold enrichment of 6005X (mean TAAR fold enrichment = 11320X) (Fig. 1b, c). Spearman’s rho for OR and TAAR transcript abundances between uncaptured and captured samples was 0.71 (*P* < 2.2 × 10^−16^) (Fig. 1b). Further, four sets of independently captured technical replicates from two different OBs (two distinct spike-in mRNAs for RNA subsamples from each of the two bulbs with two technical replicates per subsample) exhibited a Spearman’s rho of 0.95 (*P* < 2.2 × 10^−16^) (Fig. 1d and Fig. S1d). The fold enrichment and correlation between pre- and post-capture samples indicates the targeted capture approach enriches the majority of ORs and TAARs in a highly consistent fashion as evidenced by the technical replicate correlation. The mean pairwise Spearman’s rho for three biological replicate OBs was 0.83 (*P* < 2.2 × 10^−16^), indicating the relative abundance of OR and TAAR transcripts is conserved between individual animals (Fig. 1e and Fig. S1e).

In summary, targeted capture consistently enriched OR and TAAR transcripts to levels that facilitate positional analysis. This encouraged us to conduct targeted capture of ORs and TAARs from sequential sections of OB to determine which ORs were expressed in each section. We sectioned from three directions, dorsoventral (DV), anteroposterior (AP), and mediolateral (ML). We note that these three axes are not precisely orthogonal and not perfectly concordant with the corresponding reference features such as DV zonal boundaries and the medial surface of the OB.

### Expression of OR and TAAR mRNA in dorsoventral OB sections correlates with OE expression zones

Pioneering investigation established that OR transcripts are expressed in overlapping, continuous zones of the OE along the DV axis^35,36^. This zonal OE organization further correlates with DV glomerular positions of OR mRNA expression in the OB such that OSNs located in the dorsal OE target to the dorsal OB and ventrally localized OSNs target to the ventral OB^5,7,23,26,27,29,37–40^, with more recent studies leveraging multiplexed assays and transcriptomics to map an expanded number of OR transcripts to more specific OE zones^41,42^. To comprehensively assess the relationship of OE-OB DV zonal organization of OR mRNA expression, we collected 100 μm sequential sections along the OB DV axis for targeted transcriptomics to determine which OR transcripts were expressed in each section. Canonically, each OR mRNA is expressed in two glomeruli per bulb, both of which are expected to be in similar positions along the DV axis. If enriched OR sequences are from OSN axon terminals, we expect that expression of each OR transcript would be abundant in spatially clustered sections which reflect the OE DV position from which the axons originate.

**Fig. 2:**
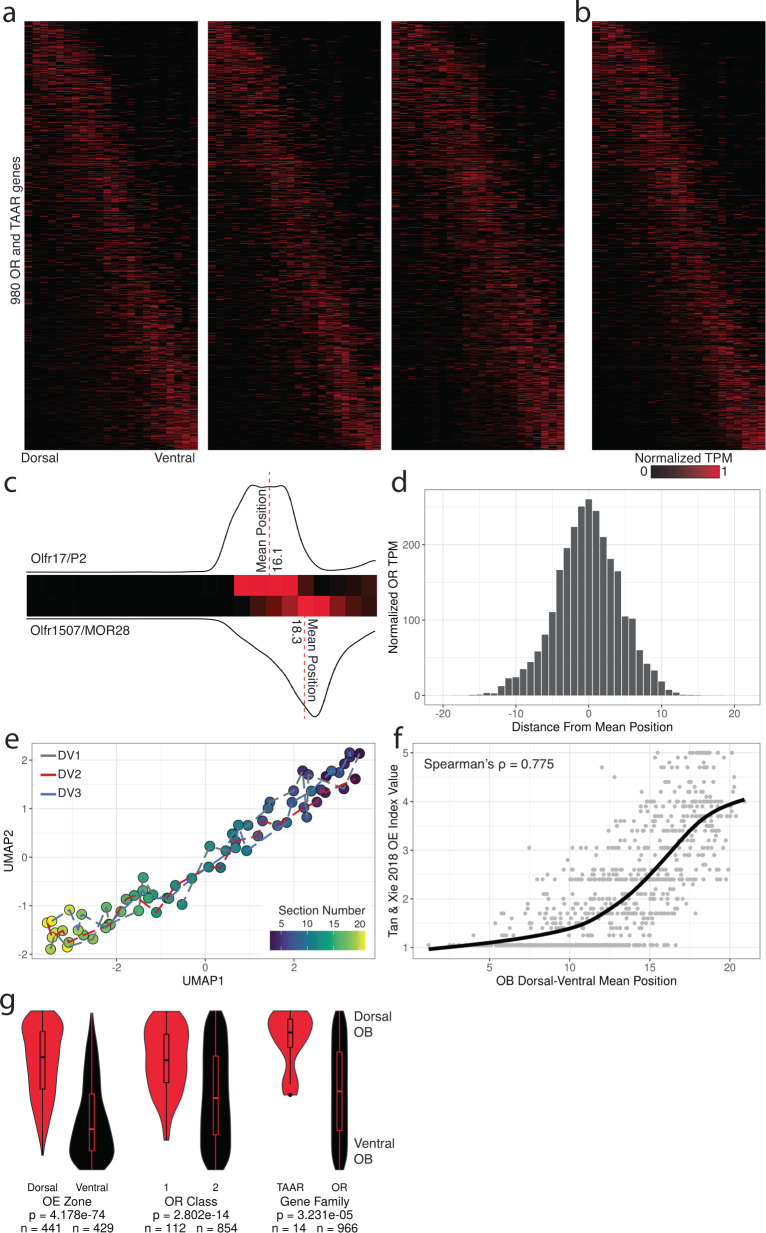
Glomerular OR expression correlates with OE expression along the DV axis. **a** Heatmaps for expression of 980 OR and TAAR genes across 22 DV sections sorted by mean position of expression from three replicate mice. Source data provided as a Source Data file. **b** Merged representation of DV replicates. Ordering of genes (y-axis) is consistent across all heatmaps. **c** Depiction of DV mean position for two ventral OR transcripts, *Olfr17*/*P2* and *Olfr1507*/*MOR28*, based on the transcriptomic weighted average of position from an individual replicate dataset. **d** Distribution of normalized TPM (maximum observed value =  1, minimum observed value = 0) for all 980 OR and TAAR mRNA from position of mean expression. **e** UMAP projection of 22 DV sections from all three replicates. **f** Loess-smoothed regression of OE DV index from Tan and Xie, *Chem. Senses* 2018 across DV mean positions from our targeted spatial data. **g** Distribution of ranked DV mean positions for the 980 OR and TAAR transcripts by OE zone, OR class, and gene family. Statistic is Mann-Whitney U-test. Source data provided as a Source Data file.

After weighting and normalization across individual mice, the localization pattern of each OR and TAAR transcript was limited to a single spatial cluster in a series of neighboring sections along the DV axis for a majority of the capture-enriched transcripts (Fig. 2a, b, d). Uniform Manifold Approximation and Projection (UMAP)^43^ visualization of data from the three DV replicate mice (22 sections per replicate) placed sequential sections from replicate animals in an ordered, non-clustered path, indicating that spatially-related sections have similar transcriptional profiles (Fig. 2e). Replicate heatmaps were similar to each other (mean pairwise matrix correlation RV_adj_ = 0.1803), which supports the stereotyped targeting of glomeruli to local domains^44,45^. To assess concordance of OB and OE positions along the DV axis, we constructed an expression-weighted mean DV position for each OR from the average of all three DV replicate mice, which we found correlated with the published OE DV positions of each OR (Spearman’s rho of mean position and OE index = 0.775, *P* < 2.2 × 10^−16^, Spearman’s rho = −0.690, *P* < 2.2 × 10^−16^) (Fig. 2f and Fig. S2a)^41,42^. Along the DV axis, we found dorsal OE OR, Class I OR, and TAAR transcripts primarily localized in the dorsal OB sections, while Class II OR tanscripts were distributed evenly along the DV axis, in agreement with previous mapping studies (dorsal vs ventral *P* = 4.178 × 10^−74^, Class I vs Class II *P* = 2.802 × 10^−14^, TAAR vs OR *P* = 3.231 × 10^−5^, Mann-Whitney U-test) (Fig. 2g)^29^.

### Anteroposterior spatial sections reflect stereotyped targeting of mirror-symmetric glomeruli

Using the same approach as for the DV axis, we examined 100 μm sections along the AP axis of the OB to further assess the precision and reproducibility of our method and the stereotypical patterning of OR glomeruli. Prior studies have shown that each OR typically has two associated glomeruli located in distinct, yet spatially linked AP and mediolateral (ML) positions, as each OB is organized into half bulbs along a non-orthogonal symmetry line^8,46,47^. This symmetry typically leads to more posterior positioning of the medial glomerulus for an OR. However, in cases where the target location of an OSN is close to the symmetry line, both glomeruli may appear in the same AP plane or only a single glomerulus may form^48^. Based on these studies, we hypothesized that a majority of OR transcripts would exhibit a bimodal expression pattern along the AP axis.

**Fig. 3:**
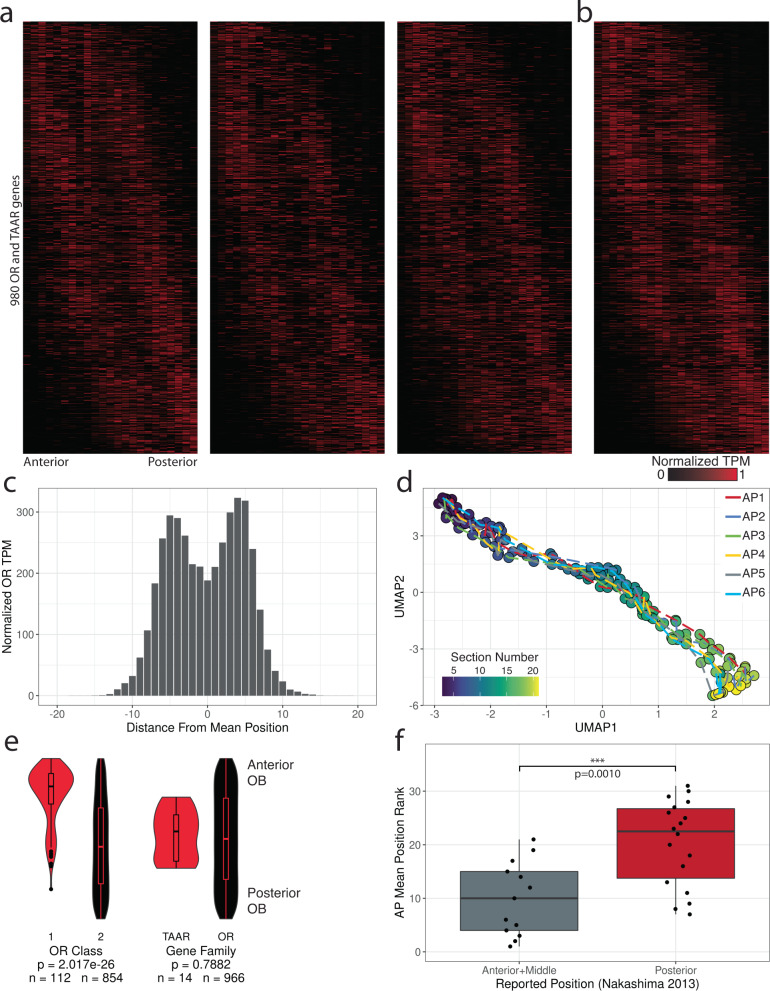
Glomerular OR gene expression is bimodally distributed along the anteroposterior axis. **a** Heatmaps for 980 OR and TAAR genes across 23 AP sections sorted by mean position of expression from three replicate mice. Source data provided as a Source Data file. **b** Merged representation from A. Ordering of genes is consistent across all heatmaps. **c** Distribution of normalized TPM (maximum observed value = 1, minimum observed value = 0) for all 980 OR and TAAR genes from position of mean expression. **d** UMAP projection of 23 AP sections across all six replicates. **e** Distribution of ranked AP mean positions for the 980 OR and TAAR genes by OR class and gene family. Statistic is Mann-Whitney U-test. Source data provided as a Source Data file. **f** Distribution of the ranked AP mean position for the set of OR genes cloned from the anterior and middle OB positions (*n* = 13) vs the posterior OB position (*n* = 18) from Nakashima et al. *Cell*. 2013. Statistic is Mann-Whitney U-test. Source data provided as a Source Data file.

Spatial expression patterns for 966 OR and 14 TAAR genes, measured by the normalized transcript abundances, were consistent across replicates with stereotyped AP glomerular positions across OBs^8,49^. When sorted by position of mean expression, each OR transcript primarily displayed two peaks of expression, consistent with published studies for labeled ORs displaying the medial glomerulus in a more posterior location relative to the lateral glomerulus (Fig. 3a, b, and Fig. S3a)^8^. Compared to the distribution of normalized OR gene expression across the DV axis, OR transcripts along the AP axis were distributed bimodally (Fig. 3c). Similar to the DV axis, UMAP projections of gene expression values from the six AP replicate mice (23 sections per replicate) revealed correlated expression patterns and AP positions across each OB (Fig. 3d). Across the AP axis, we found Class I OR mRNAs biased to the anterior set of sections, while TAAR mRNAs tended to localize to the central portion of the axis (Class I vs Class II *P* = 2.017 × 10^−26^, TAAR vs OR *P* = 0.7882, Mann-Whitney U-test) (Fig. 3e), consistent with previous studies examining glomeruli labeled in gene-targeted mice^29,50^. We also examined our data for concordance against the set of 32 OR genes cloned from the anterior, middle, and posterior sections of an OB from Nakashima et al.^51^. The OR genes cloned from the anterior and middle OB (*n* = 13) on average occupied a significantly more anterior position in our data than OR genes cloned from the posterior OB (*n* = 18) (anterior + middle OB cloned ORs vs posterior OB cloned ORs *P* = 0.001, Mann-Whitney U-test) (Fig. 3f and Fig. S4a). We further divided these cloned OR genes across dorsal OE (*n* = 11) and ventral OE (*n* = 20) zones and found that both sets displayed concordance with our AP data, with OR genes cloned from the anterior and middle OB positions having a lower AP mean position than OR genes cloned from the posterior OB (dorsal OE: anterior and middle OB cloned ORs vs posterior OB cloned ORs *P* = 0.1636, ventral OE: anterior and middle OB cloned ORs vs posterior OB cloned ORs *P* = 0.0117, Mann-Whitney U-test) (Fig. S4b, c). Our high-throughput mapping of OR transcript positions along the OB AP axis not only displays concordance with the largest available dataset of AP positions, but expands the available information from 32 to 966 ORs.

### Relationship of OR protein sequence and glomerulus position

**Fig. 4:**
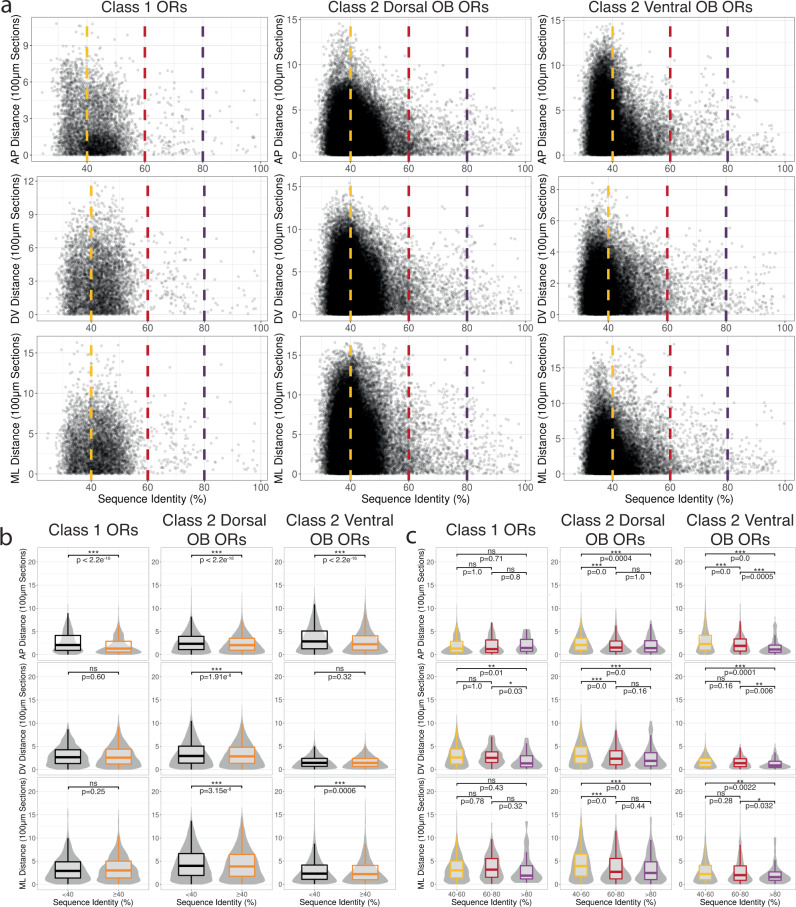
OR protein sequence similarity correlates with glomerular position more strongly among ventral than dorsal Class II ORs. **a** Scatter plot of mean position distance and OR protein sequence identity for ORs. Dashed lines indicate family (40% in yellow), subfamily (60% in red), and highly similar (80% in magenta) OR level homology cutoffs for the AP (top), DV (middle), and ML dimensions (bottom). Source data provided as a Source Data file. **b** Pairwise comparisons between mean position distance and OR protein sequence identity for ORs split by the family level homology cutoff (≥40% in orange, <40% in black) for the AP (top), DV (middle), and ML dimensions (bottom). Class 1 ORs: *n* < 40% = 1912, *n* ≥ 3444; Class 2 Dorsal OB ORs: *n* < 40% = 28499, *n* ≥ 27446; Class 2 Ventral OB ORs: *n* < 40% = 23254, *n* ≥ 9889. Statistic is Mann-Whitney U-test. **c** Pairwise comparisons between mean position distance and OR protein sequence identity for ORs split by the family, subfamily, and orthologous OR level homology cutoffs (40–60% in yellow, 60–80% in red, ≥80% in magenta) for the AP (top), DV (middle), and ML dimensions (bottom). Class 1 ORs: *n* for 40–60% = 3308, *n* for 60–80% = 110, *n* for ≥80% = 26; Class 2 Dorsal OB ORs: *n* for 40–60% = 26433, *n* for 60–80% = 848, *n* for ≥80% = 165; Class 2 Ventral OB ORs: *n* for 40–60% = 9271, *n* for 60–80% = 510, *n* for ≥80% = 118. Statistic is Mann-Whitney U-test.

Gene-targeting approaches have identified a couple of examples in which closely related ORs target to nearby glomeruli^52,53^. Our dataset with a majority of ORs assigned to specific AP positions allowed us to systematically interrogate whether OR genes with similar protein sequences exhibit similar glomerular positions by computing pairwise alignments for all ORs. To assess this relationship in all dimensions, we additionally generated a ML dataset (3 replicates, 22 sections per replicate) (Fig. S5a–e). Due to the combined presence and sequence diversity of Class I ORs, Class II ORs, and TAARs on the dorsal surface of the OB, we separated our analysis into three groups: (1) Class I dorsal OB ORs, (2) Class II dorsal OB ORs, and (3) Class II ventral OB ORs. To assess OR similarity, we calculated the percent protein sequence identity and the difference between the mean expression position values (mean position distance) for all pairs of OR transcripts along each dimension and compared the results for OR pairs using percent identity cutoffs corresponding to the level of homology used to classify OR genes as belonging to a family (40%) (Fig. 4a)^54^. When comparing OR pairs above and below the family-level threshold, we found both Class II dorsal and ventral ORs above the family level threshold displayed significant lower mean interglomerular distances across the AP, DV, and ML axes, suggesting a topological relationship between glomerular positions and family-level OR similarities for Class II ORs (Fig. 4a, b). In contrast, Class I ORs did not display a consistent relationship between protein sequence similarity and mean expression position along the DV and ML axes (Fig. 4a, b and Table S6). We further examined this relationship by comparing ORs using 60% (subfamily level ORs) and 80% (highly similar) protein sequence identity thresholds^54,55^. Comparisons between family (40–60%), subfamily (60–80%), and highly similar ORs (>80%) revealed that both dorsal and ventral Class II OR glomerular positions along all axes typically become more similar or do not change with increasing sequence similarity (Fig. 4c). Class I ORs as a whole displayed largely non-significant differences in position except for comparisons against highly similar ORs along the DV axis (Fig. 4c). Altogether our comprehensive analysis generally agrees with a model in which overall similarities of OR protein sequences influence the relative glomerular locations^27,56^.

**Fig. 5:**
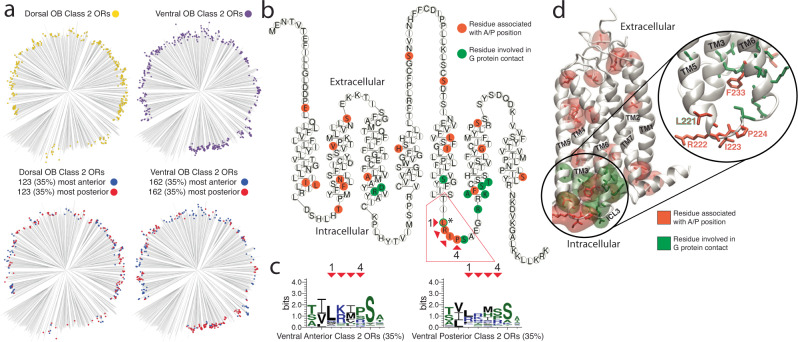
Amino acid residues associated with anterior and posterior targeting ORs. **a** Phylogenetic tree of all Class II dorsal OB OR proteins (top left, *n* = 354), all Class II ventral OB ORs (top right, *n* = 464), the most anterior (35%, blue) and most posterior (35%, red) Class II ORs from the dorsal (bottom left) and ventral OB (bottom right) DV zones. **b** Snakeplot of the Class II OR consensus protein sequence; orange residues have significantly different physicochemical properties for ventral, anterior, or posterior Class II ORs compared to all ventral Class II ORs. Green residues indicate residues known to be involved in Class A GPCR activation through contact with the G protein (* indicates the single residue which was identified as being both associated with G protein contact and identified as significantly different for ventral, anterior, Class II ORs). Source data provided as a Source Data file. **c** Protein sequence logos for the four identified intracellular loop 3 residues associated with ventral Class II anterior/posterior ORs depicting the conservation of specific amino acid residues at each position. Red arrows indicate the specific residue within the Class II OR consensus snakeplot (C) and the corresponding position in the sequence logo. **d** Homology model of the mouse Class II consensus OR protein structure. Transmembrane helices (TM) are numbered with residues associated with AP positions (orange) and residues in contact with the G protein (green) depicted with transparent regions indicating the residue surface. Right, highlight of intracellular loop 3 (ICL3) where residues associated with AP positions are intermingled with the residues in contact with the G protein.

We next sought to determine if any specific OR amino acid residues correlated with AP position. Due to the different relationships between sequence similarity and OB position for dorsal and ventral ORs, we examined Class II ORs using different cutoffs for the sets of the most anterior and most posterior ORs included in the analysis (20%, 27.5%, 35%) to identify amino acid residues correlating with AP position (Fig. 5a and Fig. S6a–c). We identified 22 residues whose physicochemical properties differed from all ventral Class II ORs (Fig. 5b and Figs. S8c and S9)^57^. Notably, four consecutive residues that correlated with AP position were in the third intracellular loop, which has been shown to interact directly with the G protein during Class A GPCR activation (Fig. 5b to d, and Fig. S8a, b)^58^. Additionally, the phenylalanine within the KAFSTCxSH motif is sandwiched between four residues involved in G-protein binding^59–61^.

In a complementary approach, we trained regression models using the XGBoost library in order to assess the predictive power of OR protein sequence on target position. Using Grantham composition, polarity, and volume values as predictors at gapless sequence for groups of ORs defined by class and dorsal/ventral expression and tested the ability of these properties to predict the position of a 10% holdout set after 10-fold cross validation. The best performing model was that of all OR sequences predicting AP mean position (Fig. S7a) (R2 = 0.74, RMSE = 1.92) but when split into dorsal and ventral OB subsets, the dorsal model had poor performance (Fig. S7b) (R2 = 0.19, RMSE = 1.87) while the ventral model was similar to that of all ORs (Fig. S7c) (R2 = 0.72, RMSE = 1.71). When we examined amino acid residues which were important to these models we found a number of positions which overlapped with our prior results including residues in the third intracellular loop. The lack of significantly associated residues and predictive power for DV and ML positions is in accordance with prior findings which have identified OR-independent factors such as chromosome location and timing of neurogenesis for positional determination along these axes^62,63^. These findings indicate residues that are at or near the sites of G-protein interactions are critical in determining glomerular position, which is consistent with the hypothesis that ligand-independent basal activity of ORs influences glomerular targeting^48,51,64–66^.

### A three-dimensional model of OR glomerular positions reflects established features

**Fig. 6:**
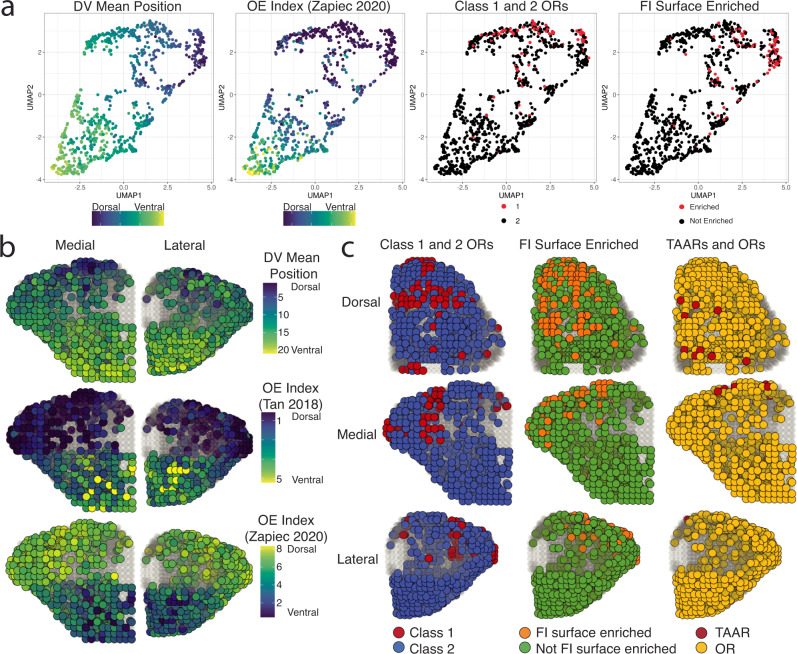
A three-dimensional model for OR glomerular positions from combined single-dimension targeted sequencing data. **a** UMAP projections of OR transcript populations constructed using the mean transcript position from each individual replicate from the AP, DV, and ML dimensions. DV mean position color reflects the calculated average mean position from all DV replicates. OE Index (Zapiec 2020) refers to the OE index established by Zapiec and Mombaerts *Cell Reports* 2020. FI Surface refers to the differential expression analysis calculated from dorsal and ventral OB samples in this paper. **b** Three-dimensional assigned positions for the 709 OR and TAAR transcripts with DV mean positions (top) and with observed values in OE DV indices (middle and bottom). Source data provided as a Source Data file. **c** Three-dimensional assigned positions for the 709 OR and TAAR transcripts with colors revealing contrasting distributions of Class I vs Class II ORs, functional-imaging-surface-enriched vs not-enriched OR and TAAR transcripts vs OR transcripts.

To date, a systematic 3D model of OR glomerular positions has yet to be made for a majority of the OR repertoire, but our high-throughput sequencing of spatial sections along the 3 cardinal axes may be combined to achieve such a result. UMAP plots of the mean position of OR trancripts in each dimensional replicate indicated that OR transcripts that are clustered on the OB surface are also clustered within the UMAP positions (Fig. 6a and Fig. S10a, b).

To account for the location of OR glomeruli on the outer surface of the OB, we extracted coordinates from a diffusion tensor imaging (DTI) model of the mouse OB to represent the approximate glomerular layer that would have been sampled by each section along each dimension of our targeted transcriptomics data^67^. We based our 3D reconstruction on a statistical approach to integrate our orthogonal sequencing data to determine which voxels on the OB surface had the highest probability for a given OR transcript^68,69^. We then developed an algorithm for the assignment of OR glomerular positions based on positional probabilities and the assumption of mirror symmetric positions producing pairs of anterolateral and posteromedial glomeruli. Lastly, we filtered out assigned positions with posterior median values below 0.0005 to account for ORs with low expression resulting in a 3D model with a theoretical spatial resolution maximum of 100 μm. Posterior median summaries for the resulting set of 709 OR and TAAR transcripts with assigned glomeruli positions as well as the raw positional probabilities for all 980 OR and TAAR transcripts in all voxels are freely viewable as interactive 3D maps at kanazian.shinyapps.io/obmap/.

**Fig. 7:**
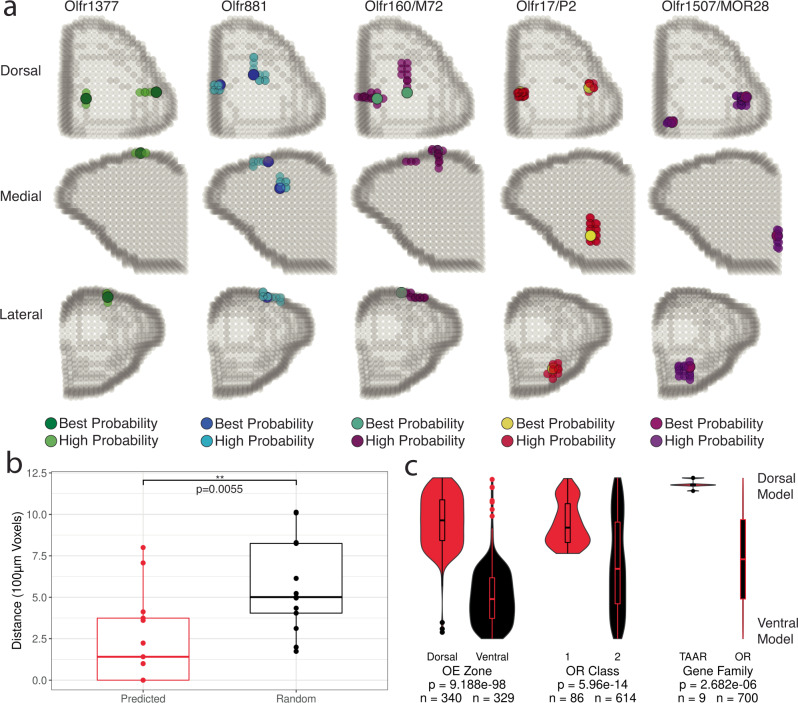
Modeled positions of glomeruli with known positions and OR transcript identity. **a** Three-dimensional assigned positions of glomeruli with labeled ORs generated in this study and from Zapiec and Mombaerts *PNAS* 2015. High probability positions indicate the set of adjacent voxels containing highly ranked probabilities for that OR with the best probability color indicating the voxel with the highest ranked probability within that cluster of voxels. Source data provided as a Source Data file. **b** Distance between assigned OR glomeruli position and OR glomeruli positions determined from gene-targeted mouse lines compared to 50 random ORs from the same DV zone (15 ORs used for unusual zone) and side of the OB. Statistic is Mann-Whitney U-test. **c** Distribution of ranked mean DV model position for the best probability voxel in each assigned glomerulus for all 709 OR and TAAR genes for OE zone, OR Class, and gene family. Statistic is Mann-Whitney U-test. Source data provided as a Source Data file.

We assessed the validity of our algorithm by comparing assigned positions for Class I and II ORs, dorsal and ventral OE ORs, and the set of ORs examined via transgenic mouse lines by Zapiec et al.^8^. Assigned glomerular positions for Class I ORs, Class II ORs, and dorsal and ventral ORs were consistent with expectations based on OE zone, OR class, and our single-dimension data (dorsal vs ventral *P* = 9.188 × 10^−98^, Class I vs Class II *P* = 5.96 × 10^−14^, TAAR vs OR *P* = 2.682 × 10^−6^, functional imaging surface enriched vs not-enriched *P* = 1.75 × 10^−24^ (see below), Mann-Whitney U-test) (Fig. 6b, c, Fig. 7c and Fig. S11b, c). Additionally, the distribution of assigned positions for the sets of Class I ORs and TAARs matched previously published findings for target domains (Fig. 6c, Fig. 11b). Our current model assigns glomerular positions for 700 ORs and 9 TAARs, with assigned positions outperforming randomly selected ORs from the same DV zone and medial/lateral side for the subset of ORs with known positions with a median error of 141 μm (Fig. 7a, b)^8^. The relative positions of the glomeruli containing *Olfr1377* and *Olfr881* transcripts  in gene-targeted mouse lines (see below) were also consistent with those assigned from the spatial transcriptomic data (Fig. 7a and Figs. S11d, S12d, S13 and S14), providing further support for our three-dimensional glomerular positions based on the spatial transcriptomic data.

Additionally we visualized the glomerular positions corresponding to ORs with shared protein sequences in the 4 amino acid long ICL3 motif identified earlier. The largest set of ORs sharing the same sequence, “LRIR”, in this motif were associated with glomeruli that displayed a distinctly anterior bias compared to a group of ORs with diverse residues at these positions but with overall similar protein sequence (Fig. S11e, f). In order to investigate the large-scale precision of the glomerular map, we constructed triplicate 3D models of glomerular positions from individual sets of AP, ML, and DV data and averaged the distance between the single best probability voxel per glomerulus between replicates in all dimensions. The median positional variance was 597 μm for 3D distance, 400 μm for AP distance, 200 μm for DV distance, and 133 μm for ML distance (Fig. S11g). The greater displacement along the AP dimension and degree of positional variance in our model agrees with prior studies^49^. In summary, we found our three-dimensional reconstruction of OR glomerular positions to be both in agreement with established OR transcript localization features and to show greater concordance with these established features than the sets of single-dimension target capture sequencing data alone. Collectively, our results thus provide the first large-scale, unified, and systematic database of OR glomerular positions for the mouse OB.

### Identification of OR transcripts within the dorsal functional imaging window

We sought to validate and extend our findings by examining specific OR transcripts that map to glomeruli on the dorsal-central OB surface, which has been extensively characterized by functional imaging in vivo^9,11,12,49,70^. To define the set of OR transcripts accessible under standard functional imaging approaches, we collected tissue samples from OBs from C57BL6 mice (2 male, 2 female). Each OB was dissected into two parts, one containing the approximate dorsal-central imaging area and the other containing the remainder of the OB (Fig. S12a). These 16 samples were processed for target capture sequencing and differential expression analysis to identify ORs enriched in the functional imaging area.

**Fig. 8:**
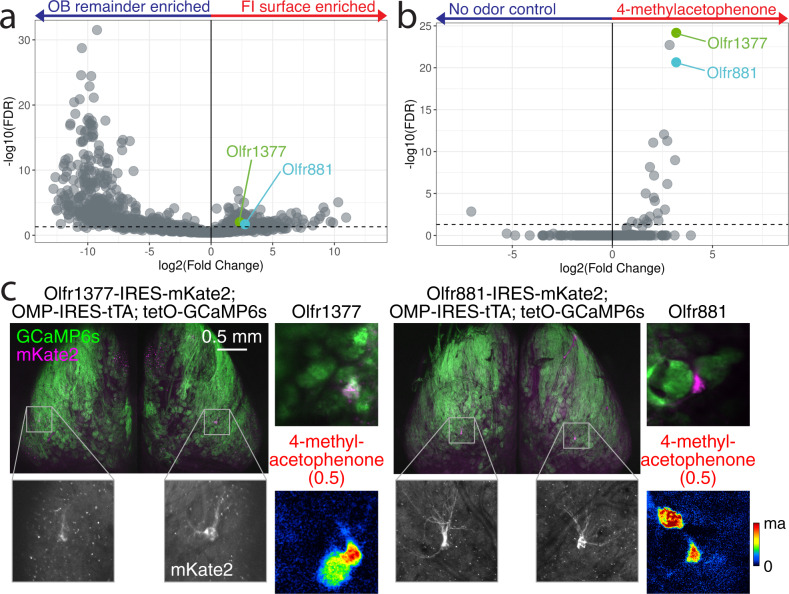
Deorphanization of Olfr1377 and Olfr881. **a** Volcano plot comparing enrichment of OR transcripts in functional imaging surface and OB remainder samples. The FI-surface-enriched OR transcripts *Olfr1377* and *Olfr881* are labeled in green and cyan, respectively. Source data provided as a Source Data file. **b** Volcano plot comparing enrichment of OR transcripts in pS6-IP RNA-Seq stimulation with 1% 4-methylacetophenone. The highly ranked responding ORs, Olfr1377 and Olfr881, are labeled in green and cyan, respectively. Source data provided as a Source Data file. **c** Whole-mount confocal maximal intensity projection of compound heterozygous Olfr1377-IRES-mKate2; OMP-IRES-tTA; tetO-GCaMP6s and Olfr881-IRES-mKate2; OMP-IRES-tTA; tetO-GCaMP6s mice following two-photon functional imaging. Baseline fluorescence and GCaMP6s ΔF/F responses to 2-s presentation of 4-methylacetophenone during two-photon functional imaging of the heterozygous lines shown on right. Estimated concentration of delivered odorant (in nM) provided in parentheses here and elsewhere. Olfr1377 response map scaled from 0–80% ΔF/F. Olfr881 response map scaled from 0–30% ΔF/F.

A total of 121 OR transcripts, including 27 Class I ORs and 94 Class II ORs, were consistently enriched in the imaging surface (FDR ≤ 0.05 and LogFC > 0) (Figs. 5b,  8a, Fig. S12, a, c, d), with 96% of these OR transcripts known to localize in dorsal OE zones^41^. We also found nine of the 15 TAAR transcripts enriched in the imaging surface, with no TAARs enriched in the remaining OB tissue (Fig. S12e), consistent with previous functional imaging of some TAAR glomeruli^50^. To anatomically and functionally validate our expression analysis, we looked to find OR transcripts which are enriched in the functional imaging surface and have a known ligand. Using the pS6-IP RNA-Seq assay in which mice are stimulated with an odor and mRNA species are immunoprecipitated from activated OSNs, we were able to define ORs which respond to 4-methylacetophenone^71^. We chose two ORs as targets for the generation of receptor-tagged gene-targeting mouse lines, based on their enrichment in the functional imaging area (Olfr881: FDR = 0.007, LogFC = 2.55 and Olfr1377: FDR = 0.021, LogFC = 2.14) (Fig. 8a) and their robust response in our pS6-IP RNA-Seq screen for ORs responding to the odorant 4-methylacetophenone (Olfr1377: FDR = 3.43e^−26^, LogFC = 3.18, Olfr881: FDR = 7.37e^−23^, LogFC = 3.18) (Fig. 8b)^71^. Using *Easi*-CRISPR^72^, we inserted *IRES*-*mKate2* cassettes following the CDS of each OR to create Olfr1377-IRES-mKate2 and Olfr881-IRES-mKate2 mice, in which OSNs expressing either Olfr1377 or Olfr881 also express the cytosolic fluorescent marker mKate2, labeling cell bodies in the OE and glomeruli in the OB (Fig. S12b). Whole-mount confocal imaging of the OB in gene-targeted mice revealed mKate2-labeled glomeruli within the dorsal-central OB surface (Fig. 8c and Fig. S13a, b).

Examination of additional whole-mount epifluorescence images allowed us to further assess position and variance of glomeruli corresponding to both Olfr1377 and Olfr881 (Fig. 7c and Fig. S14). Consistent with the expression of most ORs as two mirror-symmetric glomeruli, both gene-targeted mouse lines labeled two glomeruli per OB. The lateral Olfr1377 glomerulus (*n* = 10) was positioned centrally along on the AP axis and central-laterally within the ML axis imaging area, while the medial glomerulus (*n* = 6) was more posterior, ventral, and medial. The lateral Olfr881 glomerulus (*n* = 9) was positioned centrally along the ML axis and relatively posterior within the imaging area, while the medial glomerulus (*n* = 8) displayed a more variable position across the medioposterior quarter of the dorsal surface. The lateral Olfr1377 glomerulus displayed nearly twice (196.8%) the positional variance than the lateral Olfr881 glomerulus while the medial Olfr1377 glomerulus was distributed in an area nearly half the size (52.8%) of its Olfr881 counterpart.

### Functional imaging of dorsal OR glomeruli

**Fig. 9:**
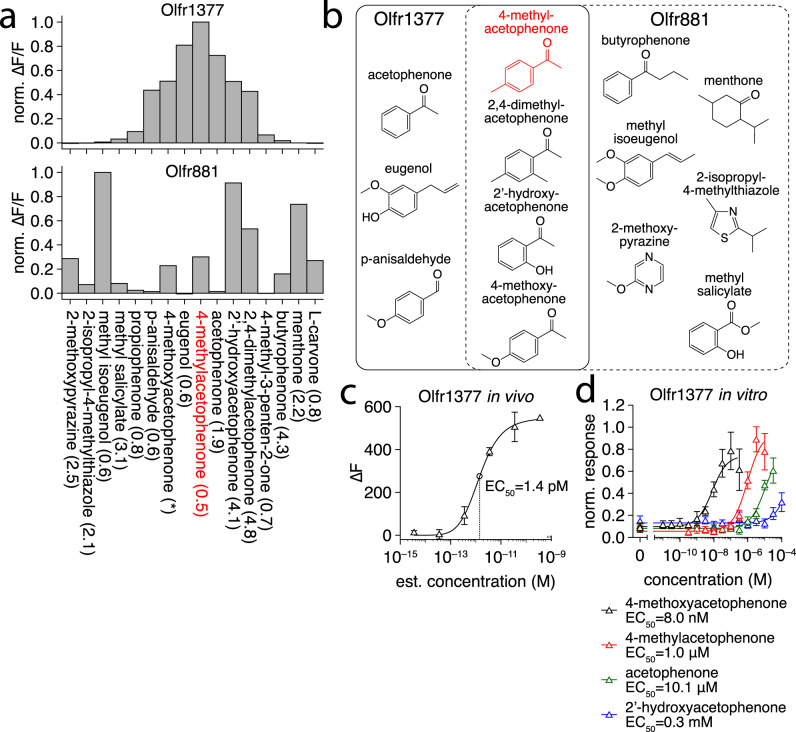
4-methoxyacetophenone is a high affinity ligand for Olfr1377. **a** Spectra of Olfr1377 and Olfr881 glomerular ΔF/F responses (sorted by Olfr1377 response magnitude) to a subset of ligands detected within a larger odorant panel. Odorants presented at an estimated concentration on the order of 10^0^ nM, with the exception of 4-methoxyacetophenone (~3.5 nM for Olfr881; ~3.5 × 10^−3^ nM for Olfr1377). Source data provided as a Source Data file. **b** Chemical structures of a subset of ligands identified by functional imaging for Olfr1377 (solid box) and Olfr881 (dashed box), including multiple overlapping cyclic ketone ligands. **c** In vivo concentration-response function of the Olfr1377 glomerulus to 4-methoxyacetophenone, *n* = 3 trials. Point and error bars represent mean and standard deviation, respectively. Source data provided as a Source Data file. **d** In vitro concentration-response function of the Olfr1377 receptor to an array of cyclic ketone odorants, *n* = 3 biologically independent replicates. Point and error bars represent mean and standard deviation, respectively. Source data provided as a Source Data file.

Expression of long-wavelength mKate2 as an OR-specific marker allowed for functional characterization of Olfr1377 and Olfr881 glomeruli by crossing the generated mouse lines to OSN-specific driver lines expressing a GCaMP Ca^2+^ reporter. For maximal imaging sensitivity, we crossed each mKate2 line to the OMP-IRES-tTA driver line^73^ and the tetO-GCaMP6s reporter line^74^. In the resulting triple crosses, we readily located the lateral mKate2-tagged glomeruli on the dorsal functional imaging surface (Fig. 8c) and imaged odorant-evoked GCaMP6s signals from these and neighboring glomeruli using dual-wavelength two-photon imaging in anesthetized mice. Consistent with our pS6-IP in vivo data (Fig. 8b), both Olfr1377 and Olfr881 exhibited robust responses to low concentrations of 4-methylacetophenone, with Olfr1377 exhibiting a stronger response than Olfr881 (Figs. 8c, 9a). In addition to 4-methylacetophenone, we also tested a large odorant panel including multiple cyclic ketones structurally related to 4-methylacetophenone, as well as more diverse odorants, all at relatively low concentrations. From this panel, we identified multiple new, high-affinity ligands for each OR, including many cyclic ketones, and ultimately uncovered overlapping but distinct response spectra for Olfr1377 and Olfr881 (Fig. 9a, b). For example, Olfr1377 showed strong responses to p-anisaldehyde, acetophenone, and the aliphatic ketone 4-methyl-3-penten-2-one, while Olfr881 proved unresponsive to these odorants.

Interestingly, Olfr1377 (but not Olfr881) exhibited an exceptionally strong response to 4-methoxyacetophenone, with a brief (2 s) presentation of ~0.4 nM 4-methoxyacetophenone eliciting long-term activation and desensitization (Fig. S15a, b). Additional concentration screening suggested an in vivo response threshold of ~10^−13^ M 4-methoxyacetophenone for the Olfr1377 glomerulus (Fig. 9c and Fig. S15c). To complement our in vivo imaging and pS6-IP RNA-Seq analyses and further evaluate the sensitivity of Olfr1377 to 4-methylacetophenone and 4-methoxyacetphenone, we additionally expressed Olfr1377 in Hana3a cells and examined luciferase responses to different concentrations of 4-methoxyacetophenone, 4-methylacetophenone, 2′-hydroxyacetophenone, and acetophenone^75,76^. Reinforcing our in vivo results, Olfr1377 responded to all four odorants (Fig. 9d). Moreover, Olfr1377 responded to 4-methoxyacetophenone at just 1 nM and saturated at 100 nM in vitro, a response orders-of-magnitude more sensitive than the response to any other odorant tested. Collectively, these findings identify 4-methoxyacetophenone-Olfr1377 as a ligand-receptor interaction with exceptionally high affinity.

## Discussion

A central goal of neurobiology is to understand how sensory systems interpret the external world^77^. The visual and auditory sensory systems feature topographic maps which organize and process stimuli such as light position and sound frequency. In vertebrates, the organization of the olfactory topographic map remains largely unknown, and understanding how OR glomeruli are organized can reveal how inputs are transformed into output signals for the olfactory cortex as well as which aspects of olfactory input are important^78,79^. In *Drosophila*, glomeruli have been individually linked to specific ORs, allowing for a comprehensive understanding of how sensory information is organized within the first olfactory relay^79–81^. This key map has served as a foundation for critical studies regarding sensory processing, olfactory receptor neuron-targeting factors, the propagation of information to higher-order neurons, and the cellular composition of the antennal lobe^82–85^.

Here, we demonstrate a unique application of target capture sequencing, enriching low-abundance target transcripts found in the axon terminals of OSNs and comparing post-enrichment abundances across samples. Our high-throughput approach enabled us to generate the first repertoire-scale dataset for OR glomeruli positions in the mouse. This approach also allowed us to create a three-dimensional positional estimate for the glomeruli of a majority of the OR repertoire with precision within the ranges previously observed for positional variability between animals^5–8,23^. Positional refinement occurs throughout the early life of the animal and is largely dependent upon intrinsic neuronal and odor-evoked activity^32,86^. We applied this approach to OBs from mice at postnatal day 21 when glomeruli targeting and formation is nearly complete. Further positional refinement at a later age would be best approached using a higher-resolution spatial method.

The rise of slide-based spatial transcriptomic technologies, such as Visium or Slide-Seq, with spatial resolutions as high as 55 μm and 10 μm, respectively, could further enhance the findings in our study^33,87,88^. These approaches can provide whole-transcriptome information enabling a greater understanding of the developmental processes within the OB. While OR transcripts in OB sections have been difficult to map to glomerular layer positions in past studies^33^, likely due to low abundance of OR mRNAs, the combination of spot-specific barcodes with targeted gene enrichment described in the current study may enable more consistent detection of these low abundance transcripts within glomeruli. Such technologies would provide histological context for specific transcripts and thus enable future studies to determinate whether OR transcripts originate from axon terminals within a glomerulus or from axon fibers extending towards a glomerulus.

Our approach to conduct 3D reconstruction is conceptually similar to that of prior bulk tissue 3D reconstruction methods^89^. To best represent our data, we embedded our method in a statistical framework that accounts for the uncertainty in count measurements and compositional limitations which underly all RNA-Seq approaches. We validated the resulting 3D model by generating and examining two new gene-targeted mouse lines for Olfr1377 and Olfr881. The observed in vivo positions and assigned 3D model positions for glomeruli corresponding to these two ORs fell within the overlapping ranges of variance for our model’s precision and the known variability in glomeruli targeting. While the use of CRISPR-based gene knock-in animals such as the ones presented in the current study will likely increase the number of gene-targeted OR lines available for study in the near future, single-molecule fluorescence in situ hybridization (smFISH) approaches are poised to rapidly and expansively allow for validation of a large number of glomeruli positions^90^. Such smFISH approaches could assess the degree and extent of positional variation among individuals when applied to serial spatial sections with multiplexed probes, or to provide direct reference positions in combination with spatial transcriptomics methods^91^.

With a systematic, anatomical mapping of OR identities to OB glomeruli, we can begin to understand which features of sensory information impact organizational strategies utilized within the mouse OB, the central circuit of olfaction. We identified functional odor-OR relationships at the peripheral interface using the in vivo pS6-IP RNA-Seq deorphanization approach. We identified a set of amino acids that included a consecutive stretch within the third intracellular loop which are correlated with anteroposterior position. This finding provides support at the repertoire level for OR targeting along the anteroposterior axis by OR basal activity in which the specific ICL3 residues may influence glomerular position by modulating G-protein contact. By identifying the set of ORs within functionally imaged glomeruli, future studies will be able to determine how OB circuits transform peripheral input into neural representations of odors at the OR level. Additional gene targeted mouse lines amenable to functional imaging will also enable us to better understand interglomerular connectivity and how patterns of inhibition are established between nearby glomeruli. The combination of our newly generated OR-OB positional data with ligand-receptor deorphanization data will therefore enable large-scale investigations into how the odor space relates to topographical features in the OB and will facilitate the development of unified, systematic models for how odorants are processed, interpreted, and transformed into behavior.

## Methods

All procedures were performed following the NIH *Guide for the Care and Use of Laboratory Animals* and were approved by the University of Utah Institutional Animal Care and Use Committee and Duke Institutional Animal Care and Use Committee.

### Animals

Wild-type male and female C57BL6 mice were purchased from the Jackson Laboratory and used for breeding. Male and female Olfr1377-IRES-mKate2 and Olfr881-IRES-mKate2 mice were generated by inserting *IRES*-*mKate2* cassettes downstream of the CDS of each OR using *Easi*-CRISPR^72^. The Olfr1377-IRES-mKate2 and Olfr881-IRES-mKate2 strains will be submitted to Jackson labs and pending approval, released for public availability. OMP-IRES-tTA mice^73^ were provided by C. Ron Yu (Stowers Institute, Univ. of Kansas). tetO-GCaMP6s mice^74^ were purchased from the Jackson Laboratory (Stock No. 024742). Mice were group housed with food and water available ad libitum and kept on a 12-h-light/dark cycle under standard temperature and humidity conditions. All procedures for animal handling and tissue collection were approved by the Institutional Animal Care and Use Committees of Duke University and the University of Utah.

### Sample acquisition and sequencing library preparation

Pups were sacrificed between postnatal day 20 and 22. Whole brains with olfactory bulbs intact were dissected and placed in a solution of 3% Low Melting Point Agarose (American Bioanalytical) within an embedding mold (Polysciences Inc, Peel-A-Way R30) before placing on ice. Once solidified, the mold was removed and the agarose-embedded brain was prepared for vibratome sectioning by cutting away agarose to leave a triangular shape with the tip forming at the bulb on the surface that will be cut first for that specific direction. The triangular block was glued (Loctite 404) onto the vibratome stand (Leica VT1000S with Feather carbon blades), submersed in cold 1X HBSS (Gibco) and 100 μm sections serially cut. Sections were placed into 1.5 mL tubes and stored at −80 C. 400 µL of Buffer RLT (Qiagen) with 10% 2-mercaptoethanol was added to each sample prior to homogenization for 4 s at 30,000 rpm using a mounted Biogen PRO200 with 5 mm flat tip generator probe. The homogenizer probe was rinsed three times with deionized water between samples. RNA was extracted using a Qiagen RNeasy kit according to the manufacturer’s instructions. cDNA synthesis was performed using a SMART-Seq v4 (Takara) kit with 10 ng total RNA used for input in half-sized reactions. 10 ng cDNA was used in the KAPA Hyperplus library construction kit following the manufacturer’s instructions. All quantifications were performed using Qubit.

### Target capture sequencing and alignment

We selected a set of ORs, TAARs, V1Rs, and V2Rs for inclusion in our assay based on the mouse genome annotation available at the time (GRCm38.p4 release M10). This set of transcripts was submitted to Roche Nimblegen for inclusion in a targeted enrichment probe panel. All sections from a single OB were uniquely indexed and combined in equal amounts to create a 1000 ng pool which was processed for target capture according to the manufacturer’s protocol using a 20-hour hybridization period. Briefly, the cDNA pool is combined with blocking oligos and biotin labeled single-stranded DNA target probes. Following the 20-hour hybridization period, the sample undergoes a series of washes before purification and elution using streptavidin conjugated paramagnetic beads resulting in a pool of target enriched libraries. Target capture library pools were sequenced on an Illumina NextSeq 500 Sequencing System in the 75SR or the HiSeq2500 in 50SR mode at the Duke Center for Genome and Biology shared core facility. Snakemake v3.5.5^92^ was used to process read files through alignment and quantification. STAR v2.7.0d^93^ was used to generate a genome index using the primary assembly and comprehensive gene annotation file of the mouse genome (Gencode GRCm38.p6 release M25). Reads were aligned to this genome index using STAR with default options except for –quantMode TranscriptomeSAM which maps genome alignments to transcript coordinates. STAR output transcriptome SAM files were quantified using RSEM v1.3.1^94^ using default options.

### Sample normalization

TPM values for each sample from each replicate OB were weighted by a factor that accounts for the proportion of OMP found in each sample and the approximate proportion of the OB glomerular layer collected in the sample (see below: Three-dimensional model of OR glomeruli positions). Weighted TPM was then normalized between 0 and 1 using the minimum and maximum values for each OR. Merged heatmaps were generated using the median normalized values at each position of the expression array for DV and ML heatmaps (*n* = 3). For AP heatmaps, the merged representations was generated from the median of four individual replicates along with the mean value of the four replicates at each position. Matrix of median values were then renormalized for each OR across samples. The following ORs were excluded from all analyses due to abnormally high expression values that potentially indicate ectopic expression of the OR within OB cell types: Olfr287, Olfr32, Olfr361, Olfr1033.

### Sequence and position analysis and identification of anterior and posterior OR associated residues

Mouse OR protein sequences were downloaded from HORDE^95^ and OR pairwise alignments were computed using the pairwiseAlignment and pid functions from the R package Biostrings using the BLOSUM62 substitution matrix, a gapOpening penalty of 11 and a gapExtension penalty of 1. 1090 mouse OR protein sequences were aligned using Clustalx with manual adjustments as previously published^71^. To identify residues that were more conserved by the 139 (30% set) most anterior, ventral, Class II ORs than by chance, we simulated distributions of Grantham distances for random subsets of 70 (50% of size of set) ventral Class II ORs for all pairwise combinations for each residue in the alignment. Random sampling was performed 1000 times. The per-residue mean Grantham distances were computed for all combinations of the 139 most anterior, ventral, Class II ORs and used to find p-values under the null distribution after FDR correction. Alignment positions with gaps in more than 10% of OR sequences were excluded from the analysis. Analysis was repeated for all sets (20%, 27.5%, 35%) for both anterior and posterior Class II ORs. Visualization of the Class II consensus OR structural sequence were generated using code from (https://github.com/Yue-Jiang/snakeplotter). Sequence logos were created using WebLogo (http://weblogo.threeplusone.com/) using default settings.

Predictive modeling of dimensional mean position from sequence features was done using the xgboost and tidymodels packages in R. A matrix of Grantham composition, polarity, and volume values was generated for all positions in the alignment that did not display gaps. Sets of ORs were divided into stratified datasets of 90% training, 10% training with 10-fold cross validation and hyperparameter tuning for min_n, tree_depth, learn_rate, and loss_reduction. XGBoost regression models with 1000 trees were used to predict the mean position value of the holdout set and R^2^ and RMSE metrics were computed. The R package, vip was used to extract predictor importance values.

### Homology model building

The protocol follows a previously published method^61^. Aligned protein sequences of 1092 mouse ORs were manually aligned to pre-aligned protein sequences of 11 GPCRS including bovine rhodopsin (10.2210/pdb1u19/pdb), human chemokine receptors CXCR4 (10.2210/pdb3ODU/pdb) and CXCR1 (10.2210/pdb3LNL/pdb), and human adenosine a2A receptor (10.2210/pdb2YDV/pdb) using Jalview^96^. Four experimental GPCR structures (1U19, 3ODU, 2YDV, and 2LNL) were used as templates to build the Class II mouse consensus OR by homology modeling using Modeller. Five models were obtained and the one fulfilling several constraints (sufficiently large binding cavity, no large, folded structure in extracellular loops, all TMs folded as α-helices, and a small α-helix structure between TM3 and TM4) was kept for further visualization.

### Differential expression analysis

The R package, edgeR (v3.34.1), was applied to the count table for all genes in the reference^97^. Common, trended, and tagwise dispersions were estimated separately and in the sequence listed. For identification of ORs in the functional imaging surface and ORs responding to 4-methylacetophenone, results were subset to olfactory receptor genes (pseudogenes included) and FDR values were recalculated from the p values obtained by performing differential expression using all genes.

### Three-dimensional model of OR glomeruli positions

A DTI model of the mouse brain^67^ was converted from.nii to.stl filetype and loaded in Blender. An array of cubes with the number of cubes per dimension matching the number of sections collected from each dimension was stretched in each dimension to encompass the entire OB in order to account for difference in age of mice. OB and cube structures were exported as separate.ply files and imported into R where a custom script was used to determine the position of cubes which contain OB surface polygons. Additional OB surface-containing cubes were included to remove gaps, model the medial surface, and account for a 100 μm wide glomerular layer within the outer surface of the OB. The final set of OB surface-containing cubes was exported as three-dimensional coordinates for use as a scaffold for the shape of the OB glomerular layer.

The composition of glomeruli in each voxel along the OB surface was calculated as the weighted average of the composition obtained from sequencing each OB section that intersected that voxel weighted by the total number of surface voxels in each section. To account for sequencing noise, the composition of glomeruli in each sequence was estimated using a Bayesian multinomial-Dirichlet model with a Dirichlet prior parameter of 0.65 reflecting the weak prior knowledge that each section likely contained only a subset of all possible glomeruli in the OB. For each sequenced section, 1000 posterior samples from this model were obtained and used in subsequent calculations of the glomerular composition in each surface voxel. Glomeruli positions were assigned using the highest probability positions from adjacent voxels where the OR was detected. Potentially duplicated positions from 3D sectioning-reconstruction process were evaluated by scanning for a high probability posterior medial glomerulus and an anterior lateral glomerulus within the dorsoventral hemisphere that matches our single dimension DV positional data and reported OE positional data. Data was visualized using plotly.

Comparison against ORs with known glomeruli positions was performed by superimposing images of mapped positions from publications on a blank OB 3D model, adjusting the dimension of the images until the most anterior, posterior, medial, lateral, dorsal, and ventral edges of the OB surface were aligned, and recording AP, DV, ML coordinates for the boundaries of the depicted glomerulus. For each glomerulus with a known position, we calculated all pairwise three-dimensional distances between the voxels within the assigned position and the depicted positions. Reported value was the minimum distance observed with any overlap between assigned and depicted position resulting in a value of 0.

### pS6-IP RNA-Seq

Three mice of either sex aged between postnatal day 20 and 22 were used per odorant or control condition. Animals were individually habituated in sealed containers for 1 h, then transferred to a new container containing a piece of filter paper enclosed in a cassette (Tissue-Tek) spotted with 10 μl of 4-methylacetophenone diluted at 1% (v/v) in distilled water or with 10 μl of distilled water (no odor control). After 1 h of exposure, the animals were euthanized, then had their olfactory epithelium dissected in cold 25 ml of dissection buffer (1 × HBSS + Ca^2+^, +Mg^2+^ [Gibco], 2.5 mM HEPES pH 7.4, 35 mM glucose, 100 μg/ml cycloheximide, 5 mM sodium fluoride, 1 mM sodium orthovanadate, 1 mM sodium pyrophosphate, 1 mM beta-glycerophosphate). The dissected tissues were transferred to 1.35 ml of cold homogenization buffer (150 mM KCl, 5 mM MgCl_2_, 10 mM HEPES pH 7.4, 100 nM Calyculin A, 2 mM DTT, 100 U/ml RNasin [Promega], 100 μg/ml cycloheximide, 5 mM sodium fluoride, 1 mM sodium orthovanadate, 1 mM sodium pyrophosphate, 1 mM beta-glycerophosphate, 1x protease inhibitor [Roche]). Bones were removed, and the tissue was mechanically dispersed three times at 250 rpm and nine times at 750 rpm using a Glas-Col 099 C K54 Tissue Homogenizer. The homogenates were centrifuged at 2318 x g using a Heraeus Biofuge Pico for 10 min at 4 °C in a 1.5 ml Lobind tube (Eppendorf), then the supernatants were collected into a new 1.5 ml Lobind tube and combined with a mixture of 90 μl 10% NP-40 and 90 μl 300 mM DHPC (Avanti Polar Lipids). The samples were centrifuged at 18,516 x g (Heraeus Biofuge Pico) for 10 min at 4 °C, then the supernatants were collected into a new 1.5 ml Lobind tube and incubated with 20 μl undiluted pS6 antibody (Cell Signaling #5364) for 1.5 h at 4 °C under constant rotation. During the last 30 min of incubation, 100 μl Protein A Dynabeads (Invitrogen) were prepared by washing three times with 900 μl of wash buffer 1 (150 mM KCl, 5 mM MgCl_2_, 10 mM HEPES pH 7.4, 0.05% BSA, 1% NP-40). The samples were added to the beads and incubated under constant rotation for 1 hr at 4 °C, followed by four washes with 700 μl of wash buffer 2 (350 mM KCl, 5 mM MgCl_2_, 10 mM HEPES pH 7.4, 1% NP-40, 2 mM DTT, 100 U/ml recombinant RNasin [Promega], 100 μg/ml cycloheximide, 5 mM sodium fluoride, 1 mM sodium orthovanadate, 1 mM sodium pyrophosphate, 1 mM beta-glycerophosphate). The samples were moved to room temperature at the final wash, and the elution was performed with 350 μl Buffer RLT (Qiagen). The RNA present in the eluate was purified with the RNeasy Micro kit (Qiagen).

### Heterologous cell expression

Hana3A cells^98,99^ were plated on 96-well plates in MEM supplemented with 10% FBS. After 18–24 h incubation at 37 °C and 5% CO_2,_ cells were transfected using Lipofectamine 2000 (Invitrogen) with 100 ng/μL plasmids coding for Olfr1377 (5 μL/plate), muscarinic acetylcholine receptor M3R (2.5 μL/plate), RTP1S (5 μL/plate), pRL-SV40 (5 μL/plate) and CRE-luciferase (10 μL/plate). pCI vector (5 μL/plate) was used instead of Olfr1377 plasmid for the transfection of control cells. 18–24 h after the transfection, the culture medium was replaced with serial dilutions of the odorants in CD293 medium (GIBCO), then the plates were incubated for 3.5 h at 37 °C and 5% CO_2_ without the lid. Luminescence was measured using a Polarstar Optima plate reader (BMG). Luminescence values (LV) were determined by subtracting basal luminescence (of an empty 96-well plate), then dividing firefly luminescence by renilla luminescence (to control for transfection efficiency and cell viability) as in the formula: LV = (firefly luminescence-400)/(*Renilla*-400). The LV values were normalized and plotted using GraphPad Prism 9 by establishing the minimum LV = 0 and the maximum LV = 1 and using the analysis log(agonist) vs. response (three parameters). CAS numbers for tested odorants are as follows: acetophenone-98-86-2, 2-hydroxyacetophenone-118-93-4, 4-methylacetophenone-122-00-9, 4-methoxyacetophenone-100-06-1.

### Functional imaging

Compound heterozygous Olfr1377-IRES-mKate2;OMP-IRES-tTA;tetO-GCaMP6s and Olfr881-IRES-mKate2;OMP-IRES-tTA;tetO-GCaMP6s mice (aged postnatal day 45-180) of both sexes were anesthetized and prepared for functional imaging with 3 Hz artificial inhalation via a double-tracheotomy procedure^100^. Mice were initially anesthetized with intraperitoneal injection of pentobarbital (50 mg/kg) and subcutaneous injection of chlorprothixene (12.5 mg/kg). Subcutaneous injection of atropine (0.5 mg/kg) was further given to minimize mucus secretions and maintain nasal patency. A double tracheotomy was performed, and anesthesia was subsequently maintained by ~0.4–0.5% isoflurane delivered in pure O_2_ to the descending tracheal tube while artificial inhalation (150-ms duration, 300-mL/min flow rate) was continuously driven at 3 Hz through the ascending tracheal tube. Mice were then head-fixed and the bone over the dorsal OB thinned.

Imaging was conducted at 15.2 Hz using a resonant-scanning two-photon microscope (Sutter Instruments) coupled to a pulsed Ti:Sapphire laser (Mai Tai HP, Spectra Physics) tuned to 920 nm and a Fidelity-2 1070 nm laser (Coherent) and equipped with a 16X, 0.8 N.A. water-immersion objective (Nikon), with emission separated by green (520/65 nm) and red (641/75 nm) filters and collected by GaAsP photomultipliers (Hamamatsu H10770B). Odorants were presented using a custom olfactometer^100^ in pseudorandom order (except for 4-methoxyacetophenone concentration series), with 2 s delivery duration and variable inter-delivery intervals. Odorants were diluted in caprylic/capric medium chain triglycerides (C3465, Spectrum Chemical Mfg. Corp.).

For response maps and spectra (Fig. 8b, c), ΔF/F responses were calculated as the difference in mean fluorescence within 4 s following odorant onset from the 4 s preceding odorant onset (the baseline fluorescence), divided by the baseline fluorescence. For display, ΔF/F response maps were bilinearly interpolated by a factor of 2 and low-pass filtered (Gaussian, standard deviation = 1 pixel) to improve map resolution and reduce map noise, respectively. For the concentration-response function of the Olfr1377 glomerulus to 4-methoxyacetophenone (Fig. 9a), ΔF responses were calculated as the difference in mean fluorescence within 2.5 s following odorant onset from the baseline fluorescence. For the lowest three odorant concentrations (in which responses fully decayed before subsequent odorant presentations), baseline fluorescence was calculated as the mean fluorescence within the 1 s preceding odorant onset. For the highest three odorant concentrations (in which responses failed to fully decay before subsequent odorant presentation), ΔF responses were calculated using the baseline fluorescence observed during the final 3.5 × 10^−13^ M odorant presentation. All ΔF/F and ΔF responses are averaged across three odorant presentations. All analysis was performed using custom code written in MATLAB (MathWorks).

### Whole-mount visualization

Adult mice of both sexes were transcardially-perfused with PBS followed by 4% paraformaldehyde and postfixed overnight before brains were extracted. For whole-mount confocal imaging, the ventral surface of the brain was adhered to a 35 mm culture dish to limit movement, covered in PBS to maintain tissue hydration, and 10 μm step z-stacks collected using a FluoView FV1000 (Olympus) confocal microscope equipped with 5X air objective (Olympus). Image analysis and maximal intensity projections were performed in ImageJ. For mapping glomerular position from whole-mount preparations, OBs from postfixed brains were removed intact and epifluorescence images of the dorsal and, subsequently, medial surfaces of each OB captured at 5x magnification, with each surface positioned roughly perpendicular to the imaging axis. Images were stitched manually in Photoshop before registration of OBs and glomerular position mapping. Assessment of Olfr1377/881 glomerulus position between individuals was performed by aligning whole mount images using the most anterior, posterior, medial, lateral, dorsal, and ventral edges of each OB and recording the relative position of the labeled glomerulus.

### Statistics & reproducibility

All statistical tests were performed in R. Correlation effect sizes were determined using the cor.test function from the stats package. All Mann-Whitney U-tests were two-tailed and performed with continuity corrections using the wilcox.test function from the stats package with the parameter var.equal set to false. Dunn’s Kruskal-Wallis test was performed with Benjamini Hochberg false discovery rate correction using the dunn.test function from the dunn.test package. Differential expression values displayed in plots were corrected for multiple comparisons using the Benjamini Hochberg false discovery rate correction in R. All box and whisker plots display the median value as the middle line, the first and third quartile as the hinges, and the farthest values within 1.5 inter-quartile range of the first or third quartile as the middle line as the whiskers unless otherwise stated. * indicates *p* value ≤0.05, ** indicates p value ≤0.01, *** indicates *p* value ≤0.001. Randomization was not relevant for our set of experiments because the same measurements and treatments were required for all samples. Similarly all samples were required to be tested in the same fashion to define the organization of glomeruli. Blinding was not relevant to our experiments because all measurements were quantitative. For these purposes, measurements for all groups were conducted equally and all measurements are objective.

### Reporting summary

Further information on research design is available in the Nature Research Reporting Summary linked to this article.

## Supplementary information

Supplementary Information

Peer Review File

Reporting Summary

## Data Availability

The sequencing data generated in this study have been deposited publicly in NCBI BioProject under accession code PRJNA773191. Additional raw and processed data that support the findings of this study are included in the Source Data file. Mouse genomes GRCm38.p4 release M10 and Gencode GRCm38.p6 release M25 are available from publicly available Gencode (www.gencodegenes.org/mouse/). Protein structures were accessed from the Worldwide Protein Data Bank (www.wwpdb.org). Source data are provided with this paper.

## Source data

Source Data

## Code availability

Code for the project has been made publicly available at https://github.com/kanazian/obmap.
