# Supplementary material for: Decoding the olfactory map through targeted transcriptomics links murine olfactory receptors to glomeruli
Source: Nat Commun. 2022 Sep 1;13:5137. doi: 10.1038/s41467-022-32267-3 (PMC9437035; doi:10.1038/s41467-022-32267-3)
Supplement: Supplementary file 1 — Supplementary Information [file 41467_2022_32267_MOESM1_ESM.pdf]

Supplemental Figures

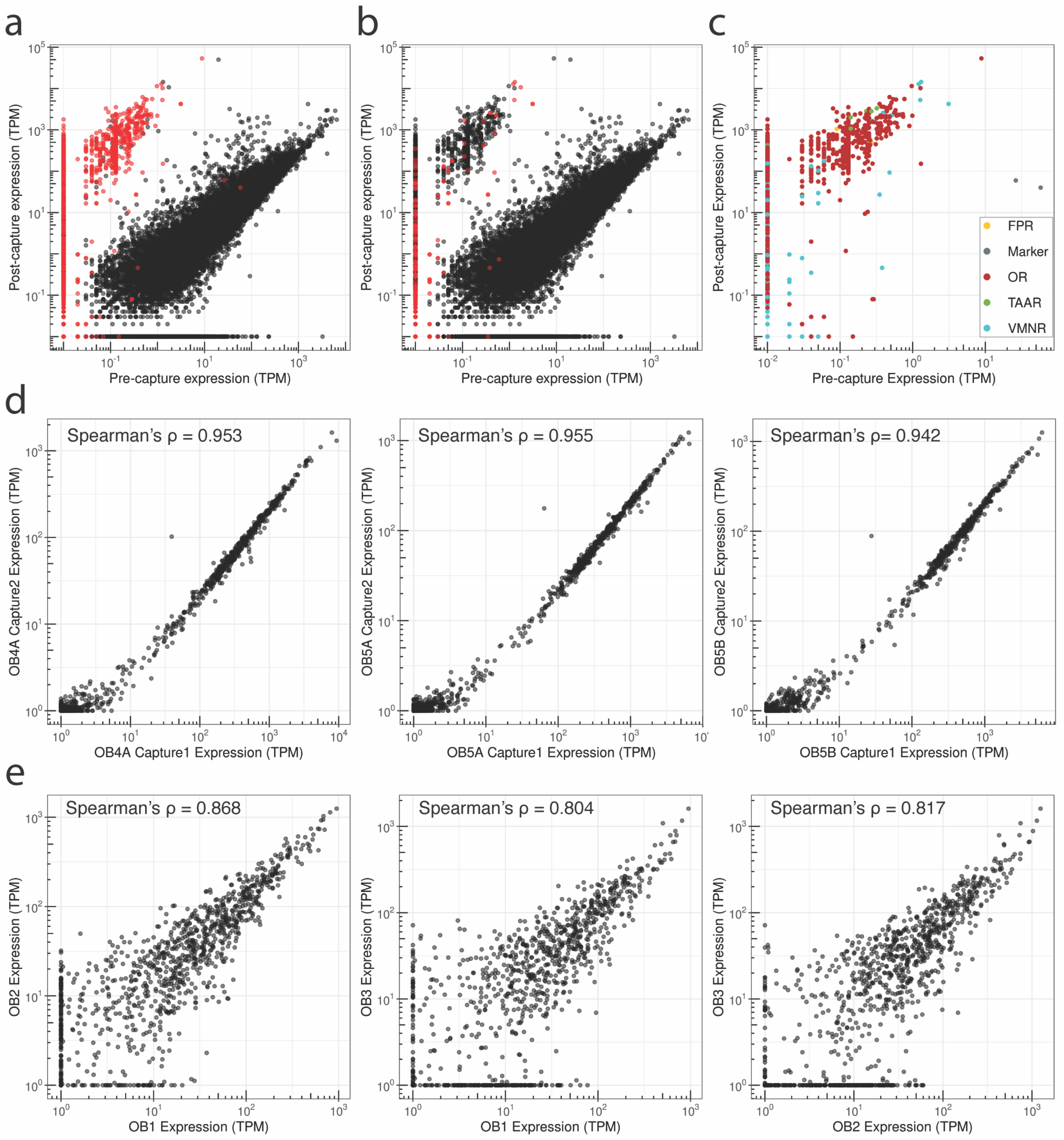

Fig. S1. Targeted capture sequencing is consistent for enrichment of OR genes. (**a**) Pre- and post-capture normalized abundances of intact probed genes (red) and intact non-probed genes (black) from a whole OB. Source data provided as a Source Data file. (**b**) Pre-

and post-capture normalized abundances of intact vomeronasal (Vmnr) genes (red) and intact non-Vmnr genes (black) from a whole OB. (**c**) Pre- and post-capture normalized abundances of target gene families. (**d**) Technical replicates of OR and TAAR gene abundances from independent capture enrichments using two different whole-OB RNA samples (OB4, OB5) combined with different ERCC spike-in mixtures (A, B). (**e**) Biological replicates of OR and TAAR gene abundances from independent capture enrichments of three different whole-OB samples (OB1, OB2, OB3).

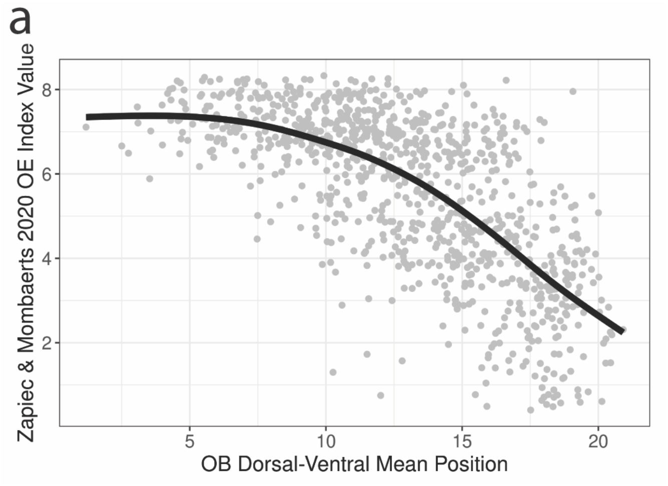

Fig. S2. Dorsoventral OB spatial sections correlate with known OE positions. (**a**) Loess smoothed regression of OE DV index from Zapiec and Mombaerts, *Cell. Reps.* 2020 across DV mean positions from our targeted spatial data.

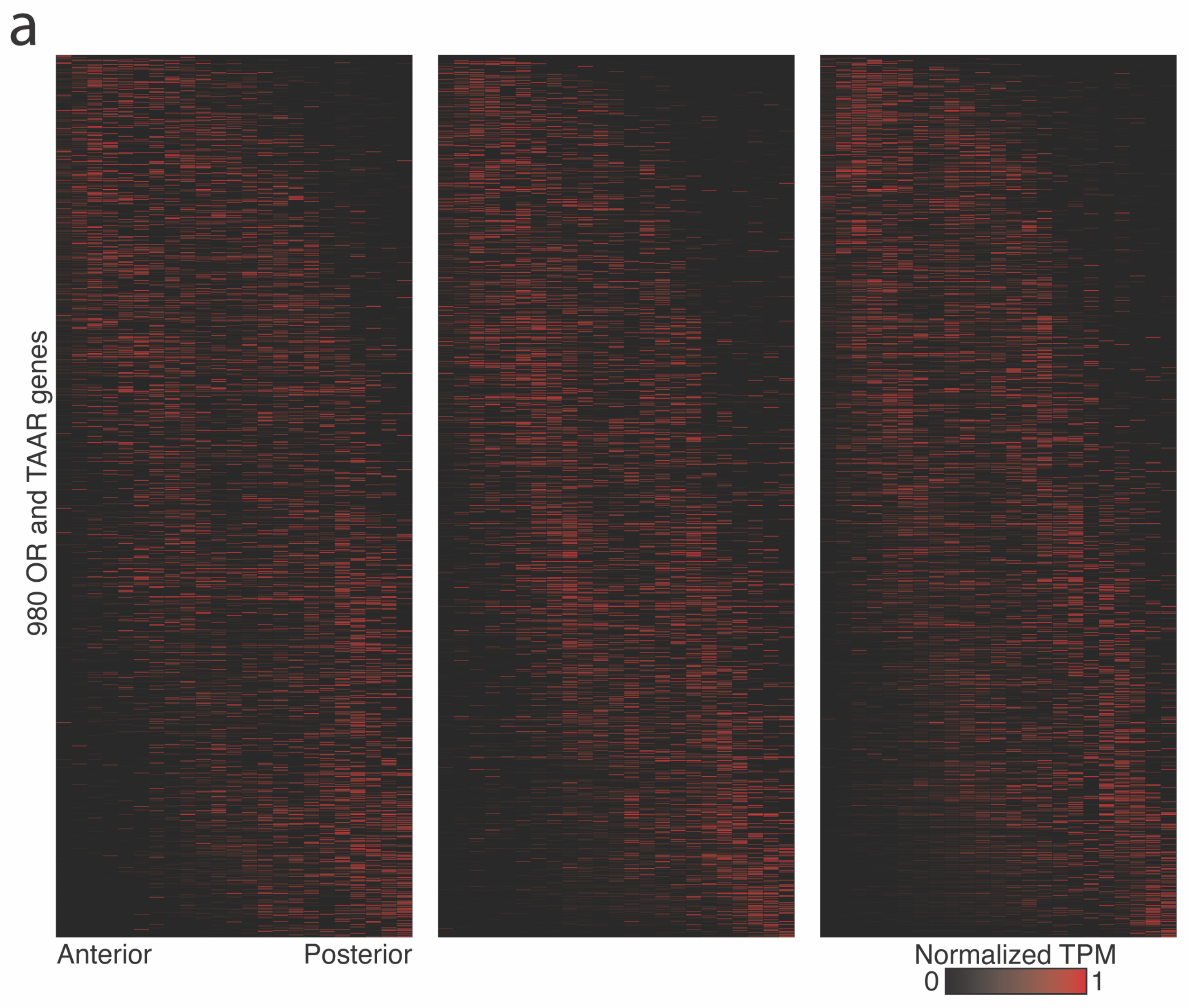

Fig. S3. ORs display bimodal distributions along the anteroposterior axis. (**a**) Heatmaps for 980 ORs and TAARs across 23 AP sections sorted by mean position of expression from three additional replicate mice. Source data provided as a Source Data file.

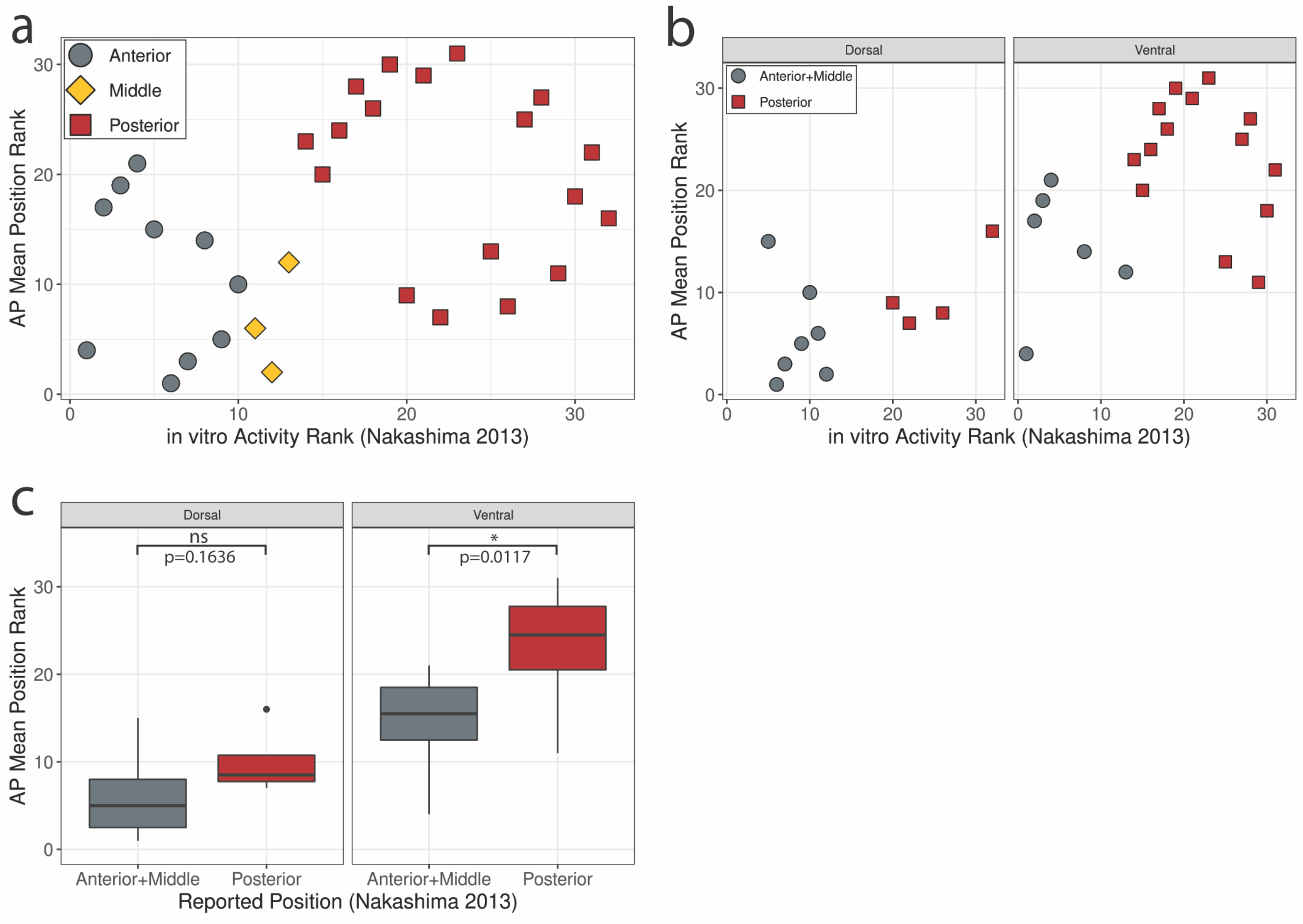

Fig. S4. Mapped AP positions are consistent with published AP data. (**a**) Scatter plot of 31 ORs present in our AP dataset examining the relationship between ranked mean AP position and in vitro activity rank from Nakashima et al. *Cell*. 2013. Color indicates the reported OB position from which the OR was cloned from. Source data provided as a Source Data file. (**b**) Data from B separated by OE DV zone. (**c**) Data from B, grouped. Dorsal Anterior+Middle n = 7, Dorsal Posterior n = 4, Ventral Anterior+Middle n = 6, Ventral Posterior n = 14. Statistic is Mann-Whitney U-test.

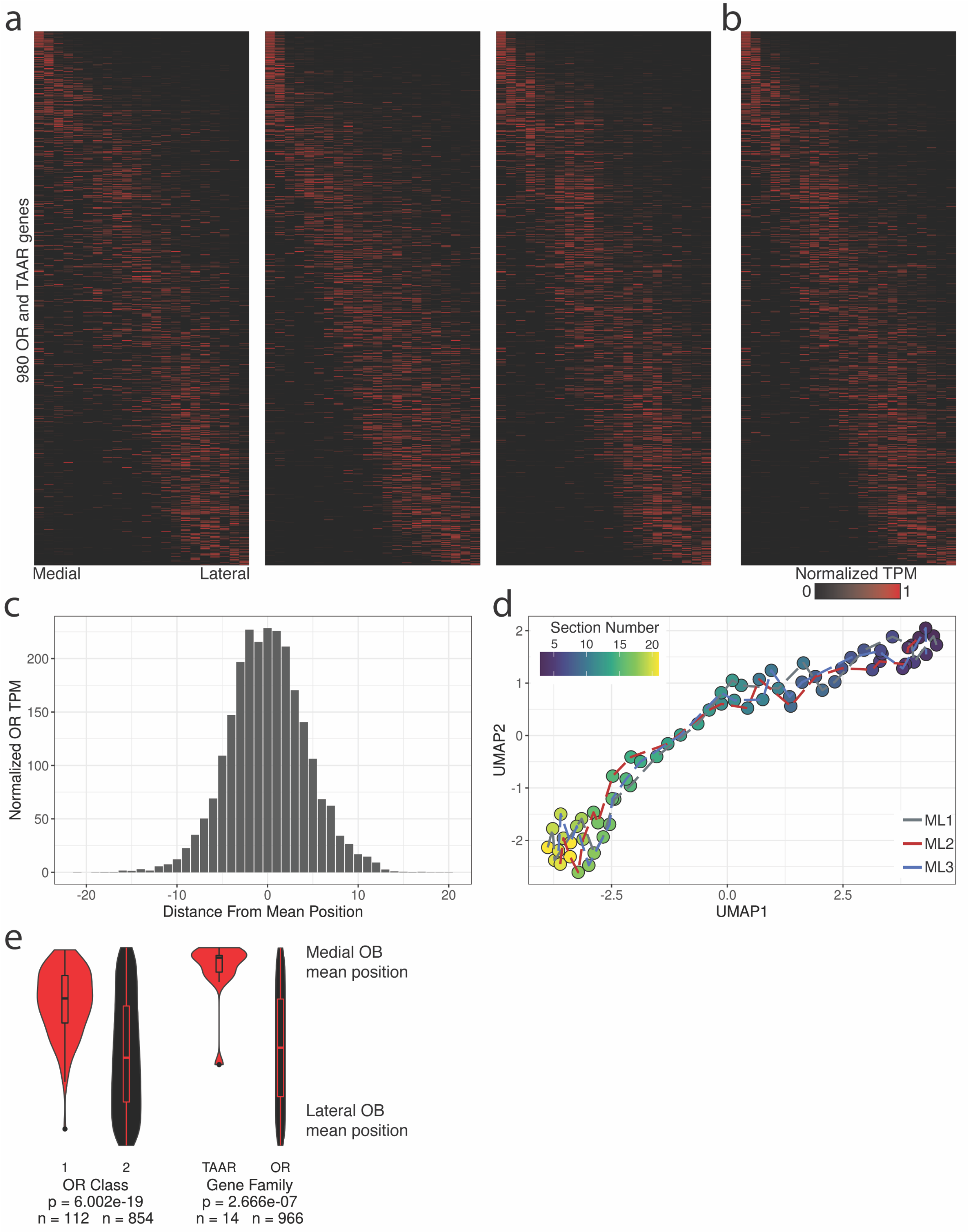
a
b
980 OR and TAAR genes
Medial
Lateral
Normalized TPM
0
1
c
Normalized OR TPM
Distance From Mean Position
d
Section Number
5 10 15 20
UMAP2
UMAP1
ML1
ML2
ML3
e
Medial OB mean position
Lateral OB mean position
1 2
OR Class
p = 6.002e-19
n = 112 n = 854
TAAR OR
Gene Family
p = 2.666e-07
n = 14 n = 966

Fig. S5. Mapping of mediolateral positions. (**a**) Heatmaps for 980 ORs and TAARs across 22 ML sections sorted by mean position of expression from three replicate mice. Source data provided as a Source Data file. (**b**) Merged representation of A. Order of Y-axis genes is consistent across all heatmaps. (**c**) Distribution of normalized TPM (maximum observed value = 1, minimum observed value = 0) for all 980 ORs and TAARs from position of mean expression. (**d**) UMAP projection of 66 ML samples from all three replicates. (**e**) Distribution of ranked ML mean positions for the 980 ORs and TAARs by OR class and gene family. Statistic is Mann-Whitney U-test.

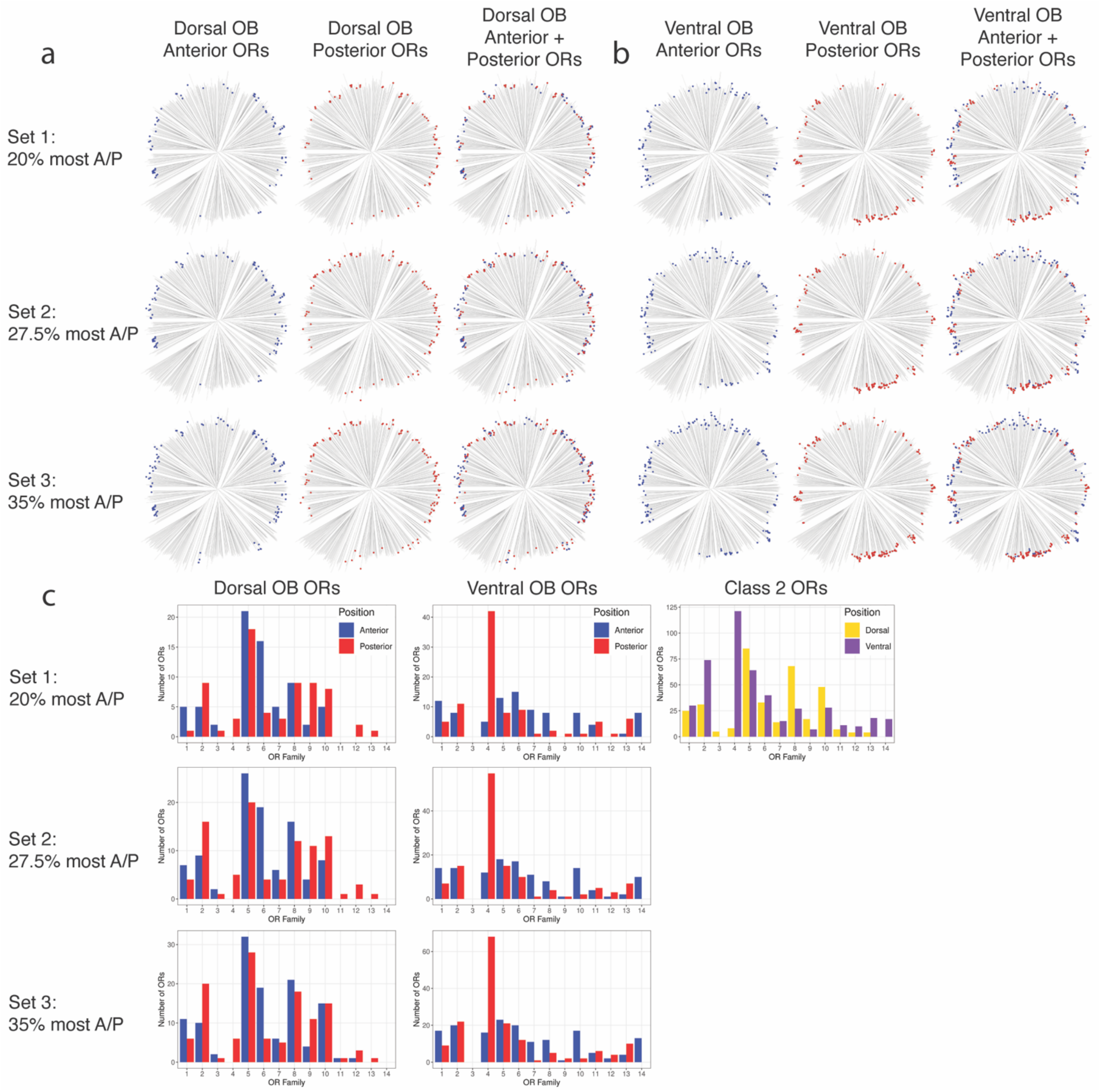

Fig. S6. Anterior and posterior ORs along the dorsal and ventral OB. (**a**) Phylogenetic trees for anterior sets of Class II dorsal OB ORs (blue, left), posterior sets of Class II dorsal OB ORs (red, middle), and combined sets of dorsal anterior and posterior Class II OB ORs (right). Sets of different sizes (top row = 20% most anterior/posterior ORs, n = 70; middle row = 27.5% most anterior/posterior ORs, n = 97; bottom row = 35% most anterior/posterior ORs, n = 123) used as conditions for identifying significantly different residues as compared to

Class II dorsal OB ORs. (**b**) Phylogenetic trees for anterior sets of Class II ventral OB ORs (blue, left), posterior sets of Class II ventral OB ORs (red, middle), and combined sets of ventral anterior and posterior Class II OB ORs (right). Sets of different sizes (top row = 20% most anterior/posterior ORs, n = 92; middle row = 27.5% most anterior/posterior ORs, n = 127; bottom row = 35% most anterior/posterior ORs, n = 162) used as conditions for identifying significantly different residues as compared to Class II dorsal OB ORs. (**c**) Distributions of dorsal and ventral anterior and posterior ORs by OR family as classified in Olender et al. *BMC Evol. Bio.* 2020 for dorsal OB ORs (left) and ventral OB ORs (middle). Distribution of all Class II dorsal and ventral ORs by OR family is depicted in the top right.

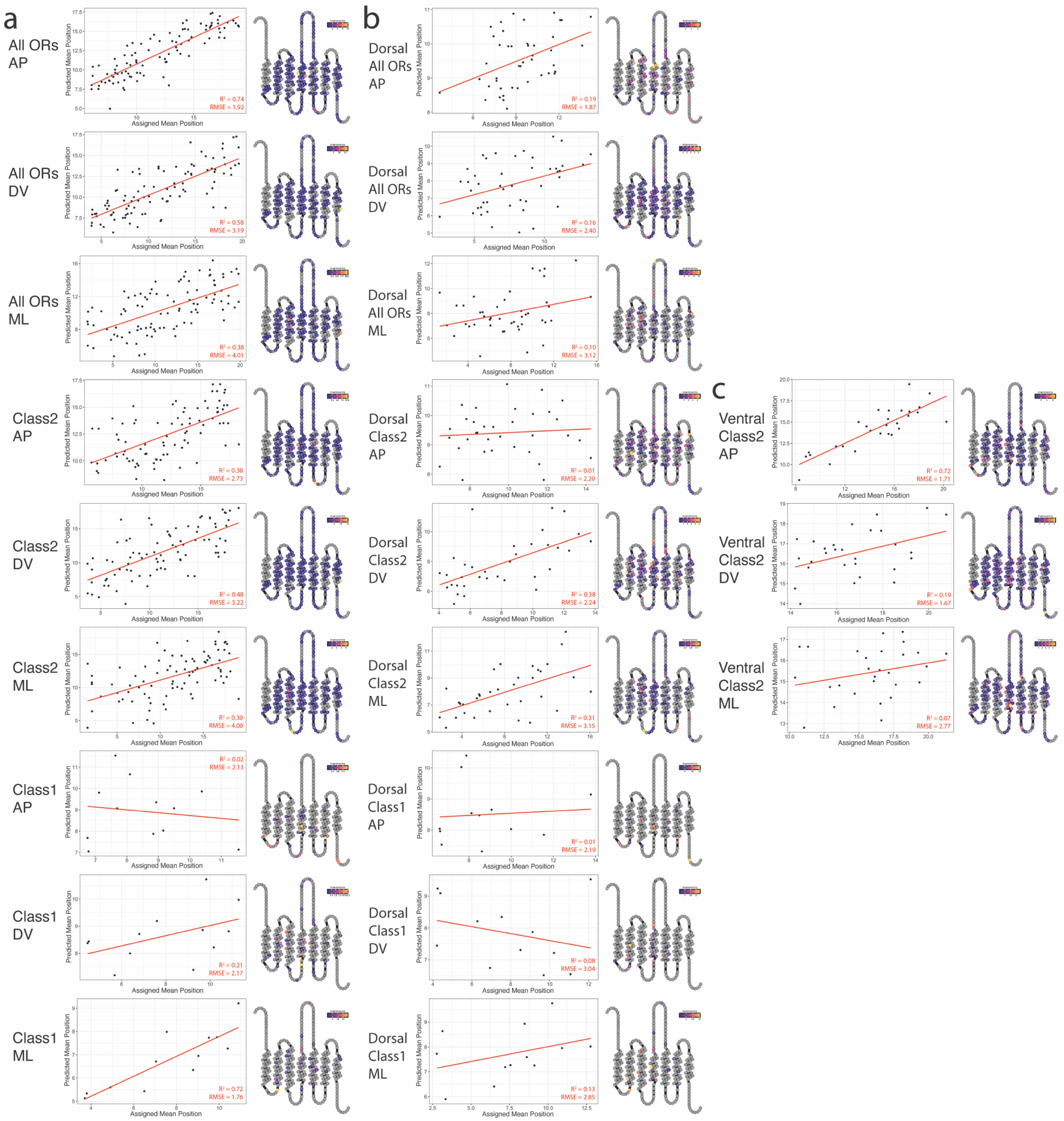

Fig. S7. Predictive power of amino acid positions on OR mean position. (**a**) Results of assigned vs. predicted mean position for holdout OR sequences using the XGBoost model with tuned hyperparameters for all ORs. All Snakeplots show aggregate importance of the composition, polarity, and volume properties for each position used by the model. Source data provided as a Source Data file. (**b**) Results of assigned vs. predicted mean position for

holdout OR sequences using the XGBoost model with tuned hyperparameters for dorsal OB ORs. Source data provided as a Source Data file. (**c**) Results of assigned vs. predicted mean position for holdout OR sequences using the XGBoost model with tuned hyperparameters for ventral OB Class II ORs. Source data provided as a Source Data file.

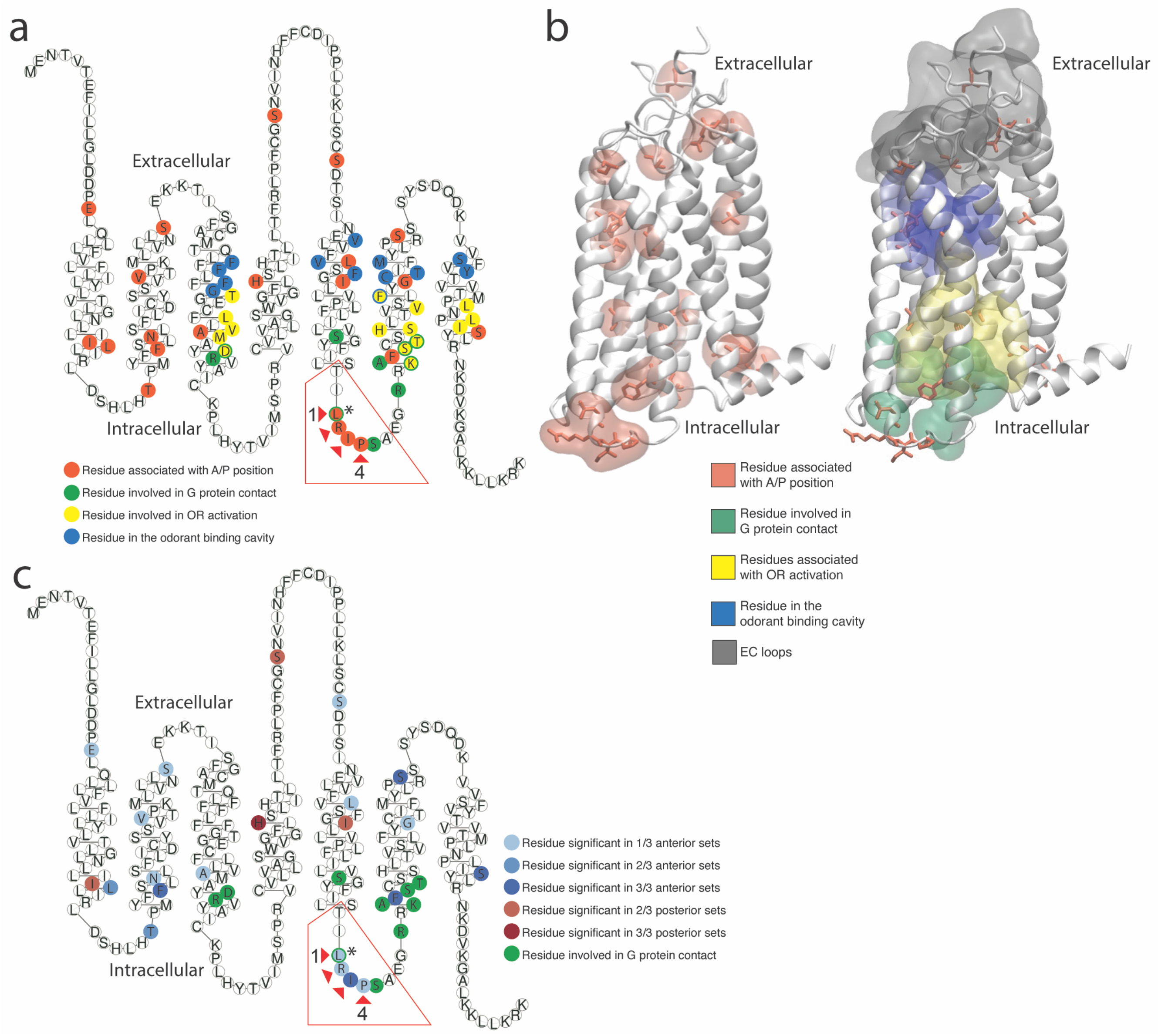

Fig. S8. Position of anterior and posterior associated residues within ventral Class II ORs. (**a**) Snakeplot of the Class II OR consensus protein sequence with residues color coded as being associated with glomerular AP position (orange), involved in G protein contact (green), involved in the OR activation mechanism (yellow), and located in the odorant binding cavity (blue). Residues filled with one color and bordered in a different color were associated with both categories. (**b**) Homology model of the mouse Class II consensus OR. Left, residues associated with AP positions (orange) are depicted in licorice with transparent

regions indicating residue surface. Right, transparent regions represent the surface of residues located in the extracellular (EC) loops (gray), located in the odorant binding cavity (blue), associated with the activation mechanism (yellow) and associated with G protein binding (green). (**c**) Snakeplot of the Class II OR consensus protein sequence with blue shaded residues were identified as having significant different physicochemical properties for ventral, anterior, Class II ORs compared to all ventral Class II ORs. Red shaded residues were identified as having significantly different physicochemical properties for ventral, posterior, Class II ORs compared to all ventral Class II ORs. Residues highlighted in green indicate mammalian OR residues known to be involved in Class A GPCR activation through contact with the G protein (* indicates the single residue which was identified as being both associated with G protein contact and identified as significantly different for ventral, anterior, Class II ORs). Source data provided as a Source Data file.

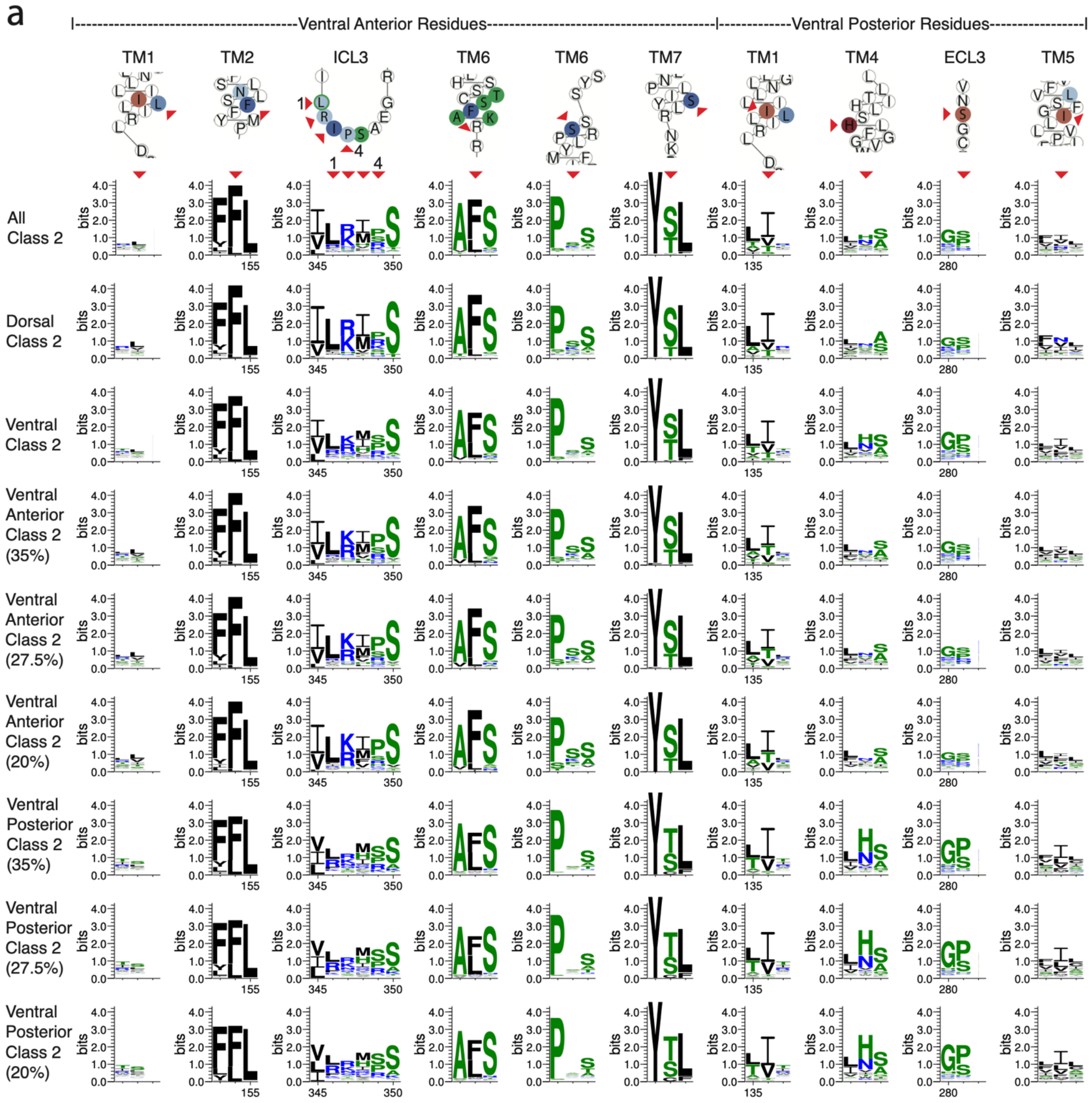

Fig. S9. Sequence logos for selected ventral Class II anterior and posterior residues. (**a**) Protein sequence logos for positions associated with ventral Class II anterior OR residues (left six columns) and ventral Class II posterior OR residues (right four columns) depicting the conservation of specific amino acid residues within different sets of sequences. Red arrows

indicate the specific residue within the Class II OR consensus snakeplot (fig. S8, a and c) and the corresponding position in the sequence logo. Source data provided as a Source Data file.

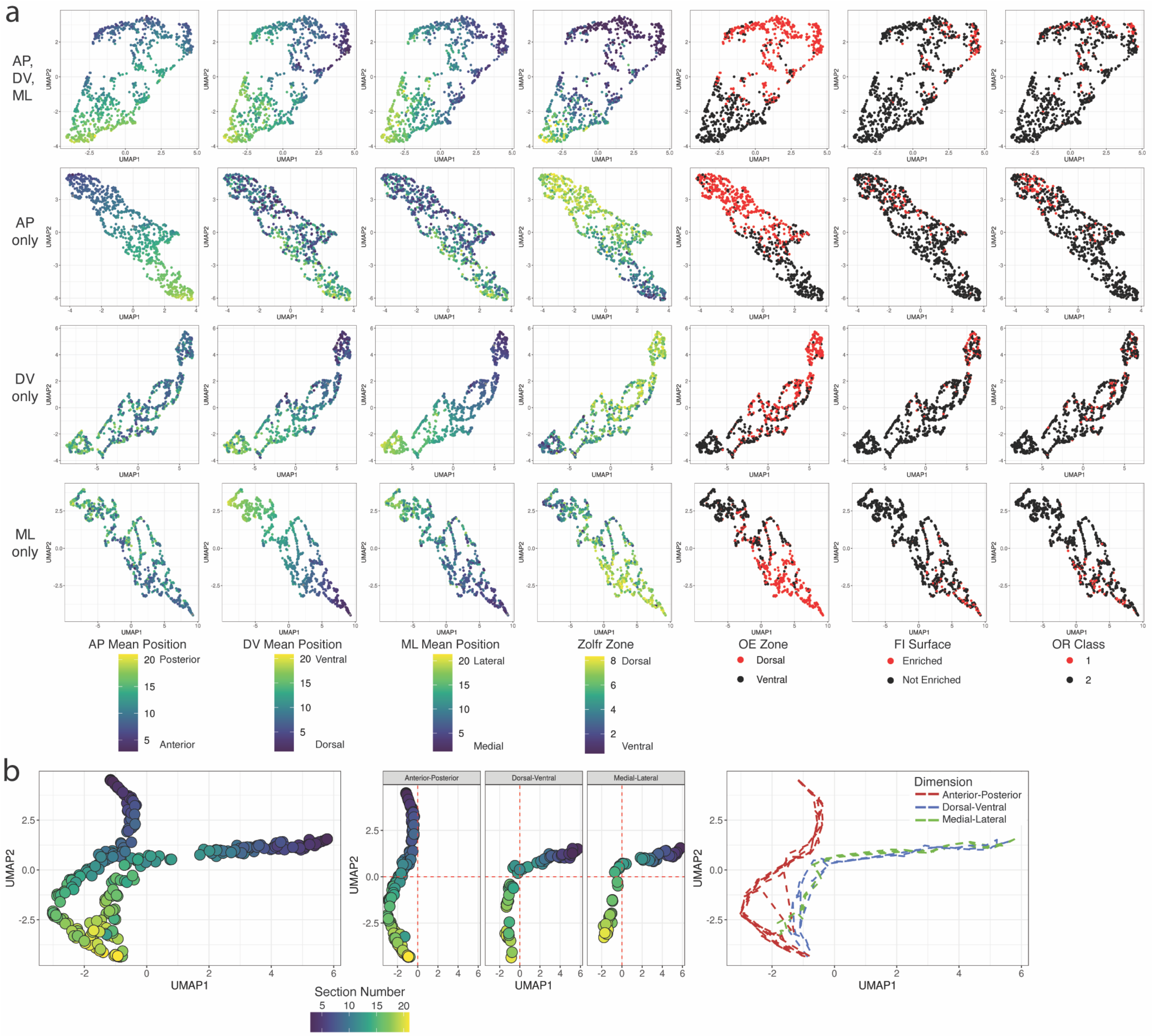

Fig. S10. Positional features are conserved when only mean position data is used. (**a**) UMAP projections of OR populations constructed using the mean position from each replicate from all dimensions (top row), AP replicates only (second row), DV replicates only (third row), and ML replicates only (bottom row). AP, DV, and ML mean position reflects the calculated average mean position from each dimension. OE Index (Zapiec 2020) refers to the OE index established by Zapiec and Mombaerts *Cell Reports* 2020. OE Index (Tan 2018) refers to the OE index established by Tan and Xie *Chem. Senses* 2018 with values less than 2 labeled as

Dorsal and equal or greater than 2 labeled as Ventral. FI Surface refers to the differential expression analysis calculated from dorsal and ventral OB samples in this paper. (**b**) UMAP projection of captured OB spatial samples constructed using transcript abundances for the 980 ORs and TAARs from all dimensions (left), separated by dimension (middle), and for the order of section number by replicate from each dimension (right).

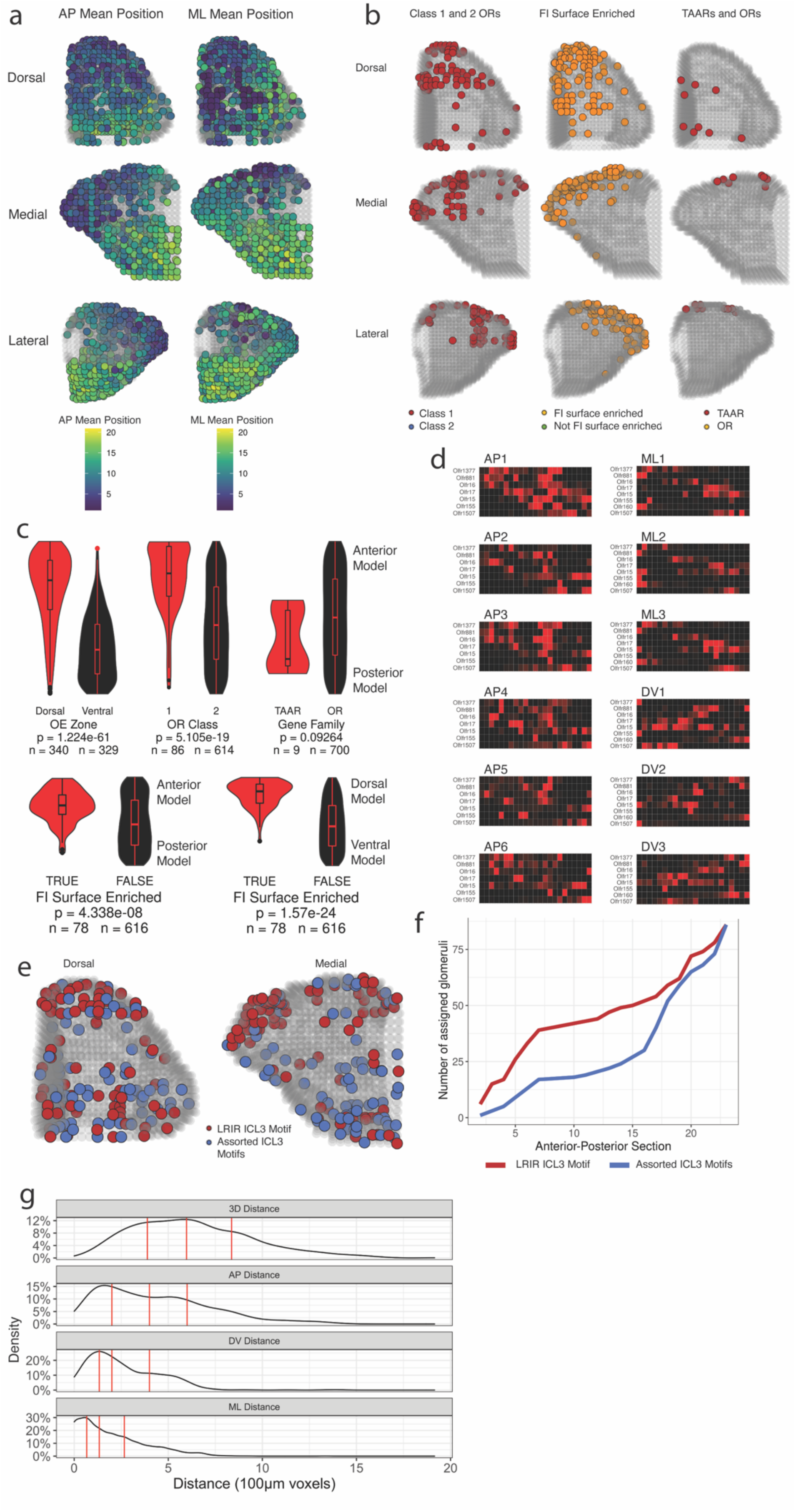
a
AP Mean Position
ML Mean Position
Dorsal
Medial
Lateral
AP Mean Position
ML Mean Position
20
15
10
5
b
Class 1 and 2 ORs
FI Surface Enriched
TAARs and ORs
Dorsal
Medial
Lateral
Class 1
Class 2
FI surface enriched
Not FI surface enriched
TAAR
OR
c
Dorsal
Ventral
OE Zone
p = 1.224e-61
n = 340 n = 329
1
2
OR Class
p = 5.105e-19
n = 86 n = 614
TAAR
OR
Gene Family
p = 0.09264
n = 9 n = 700
Anterior Model
Posterior Model
TRUE
FALSE
FI Surface Enriched
p = 4.338e-08
n = 78 n = 616
Dorsal Model
Ventral Model
TRUE
FALSE
FI Surface Enriched
p = 1.57e-24
n = 78 n = 616
d
AP1
AP2
AP3
AP4
AP5
AP6
ML1
ML2
ML3
DV1
DV2
DV3
e
Dorsal
Medial
LRIR ICL3 Motif
Assorted ICL3 Motifs
f
Number of assigned glomeruli
Anterior-Posterior Section
LRIR ICL3 Motif
Assorted ICL3 Motifs
g
3D Distance
AP Distance
DV Distance
ML Distance
Density
Distance (100µm voxels)

Fig. S11. A three-dimensional model for OR glomeruli positions from single-dimension targeted sequencing data. (**a**) Three-dimensional positional assignments for all 709 ORs and TAARs colored by AP mean position (left) and ML mean position (right). Source data provided as a Source Data file. (**b**) Three-dimensional assigned positions for the 709 ORs and TAARs colored to show the distribution of Class I, functional imaging surface enriched ORs, and TAARs without opposing features in order to better demarcate positions. (**c**) Distribution of ranked mean model position (AP axis for top row and bottom left and DV axis for bottom right) for the best probability voxel in each assigned glomerulus for all 709 ORs and TAARs for OE zone, OR Class, gene family, and enrichment in the functional imaging surface. Statistic is Mann-Whitney U-test. (**d**) Heatmaps for labeled ORs from individual replicates along the AP (left), ML (top right), and DV (bottom right) dimensions. (**e**) Dorsal and medial views of 3D positions for OR glomeruli which possess “LRIR” (red, n = 50 ORs) within the ICL3 sequence motif identified in Fig. 4 and OR glomeruli which possess a diverse set of ICL3 motif sequences (blue, n = 50 ORs). (**f**) Running aggregate of assigned glomeruli voxels by AP position. Red line represents ORs which possess “LRIR” within the ICL3 sequence motif identified in Fig. 4. Blue line represents control set of ORs which possess a diverse set of sequences within the ICL3 sequence. (**g**) Density distributions of the mean distance between the best probability voxel for OR glomeruli between individual model replicates. Red lines indicate the first quantile, median, and third quantile, from left to right.

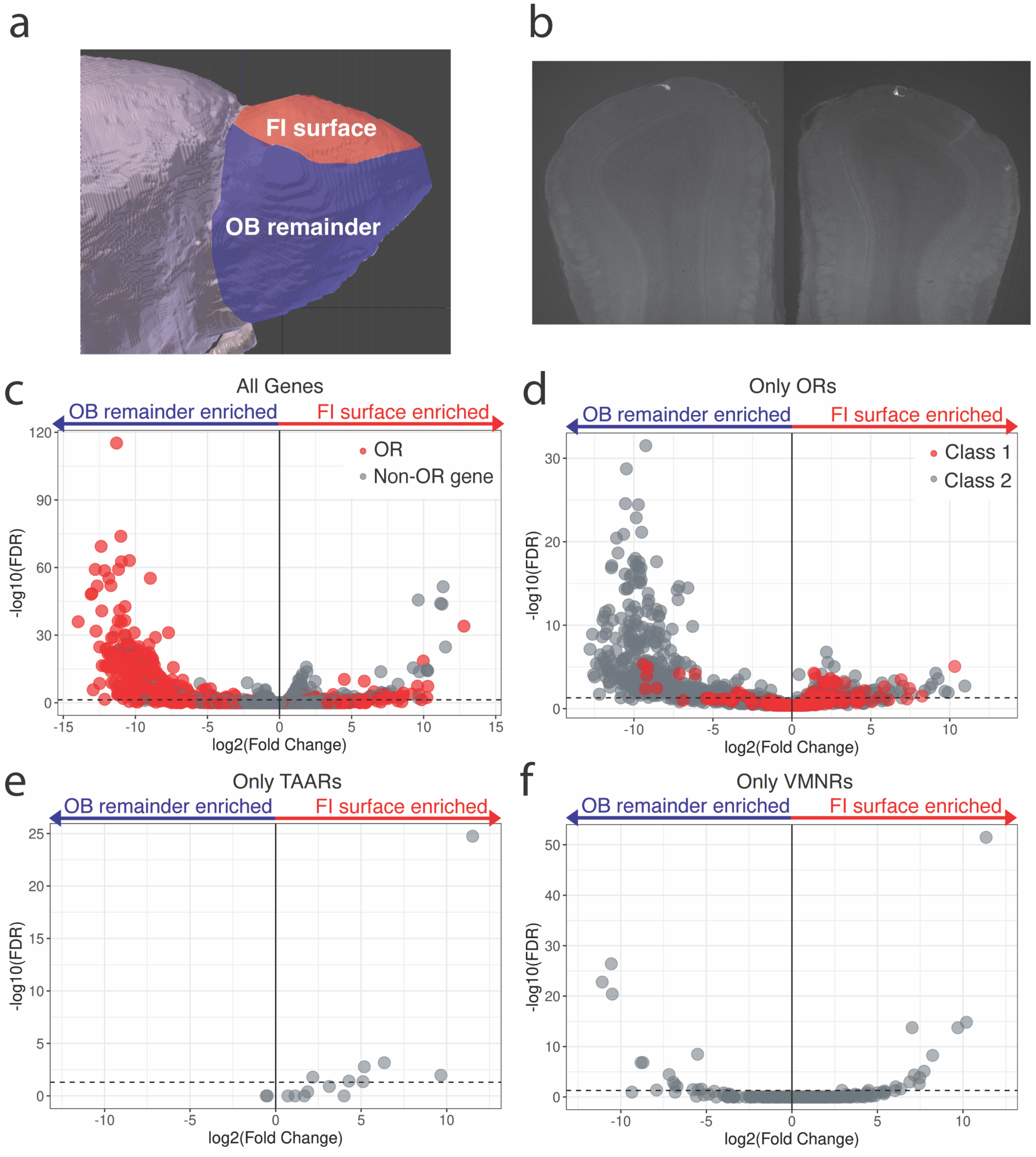

Fig. S12. Differential expression analysis for functional imaging surface samples. (**a**) OB schematic showing approximation of functional imaging (FI) surface (red) and OB remainder (blue) dissected for differential expression analysis. (**b**) Coronal sections from an

Olfr881-IRES-mKate2 mouse indicating a dorsal-central location of the mKate2 labeled ORs (**c**) Volcano plots for differential expression from functional imaging samples and OB remainder samples for all genes, ORs labeled in red. (**d**) Volcano plot for only ORs, FDR readjusted, Class I ORs labeled in red. (**e**) Volcano plot for only TAARs, FDR readjusted. (**f**) Volcano plot for only vomeronasal receptors (VMNRs), FDR readjusted.

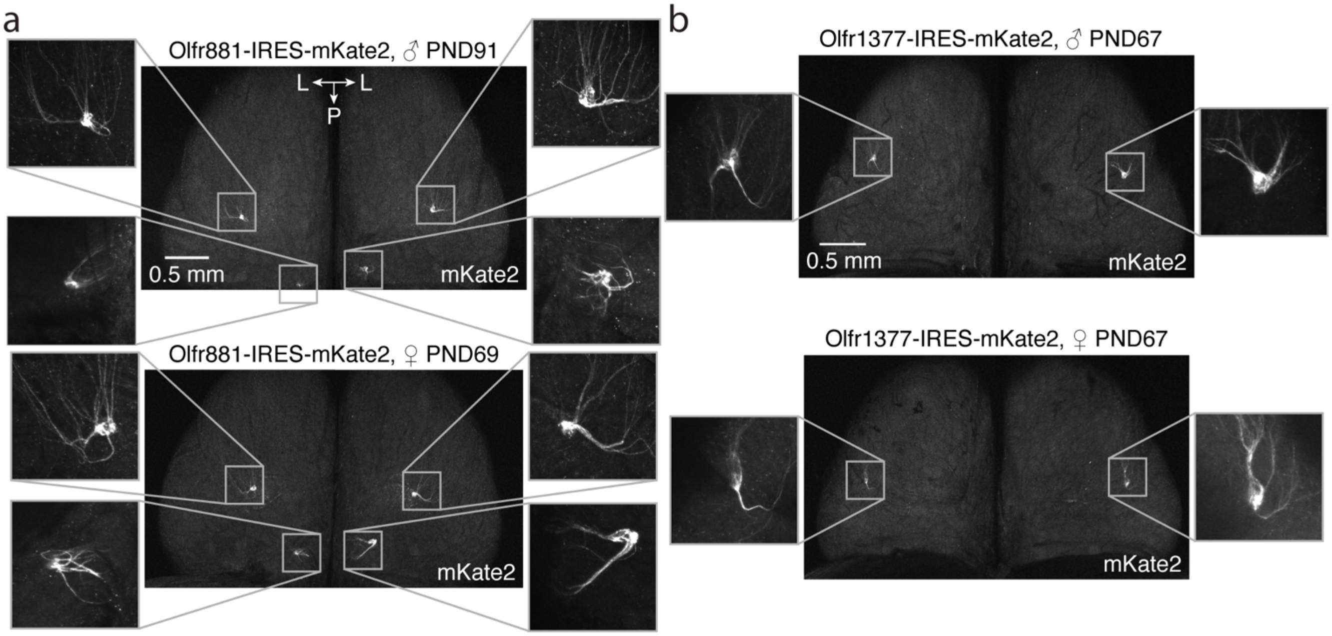

Fig. S13. Whole-mount confocal microscopy and glomerular phenotypes of Olfr1377-IRES-mKate2 and Olfr881-IRES-mKate2 OBs. (**a**) Whole-mount maximum intensity projections of the dorsal surface of Olfr881-IRES-mKate2 OBs from male and female mice with inlays showing the mKate2-positive glomeruli, n = 1 animal. (**b**) Whole-mount maximum intensity projections of the dorsal surface of Olfr1377-IRES-mKate2 OBs from male and female mice with inlays showing the mKate2-positive glomeruli, n = 1 animal.

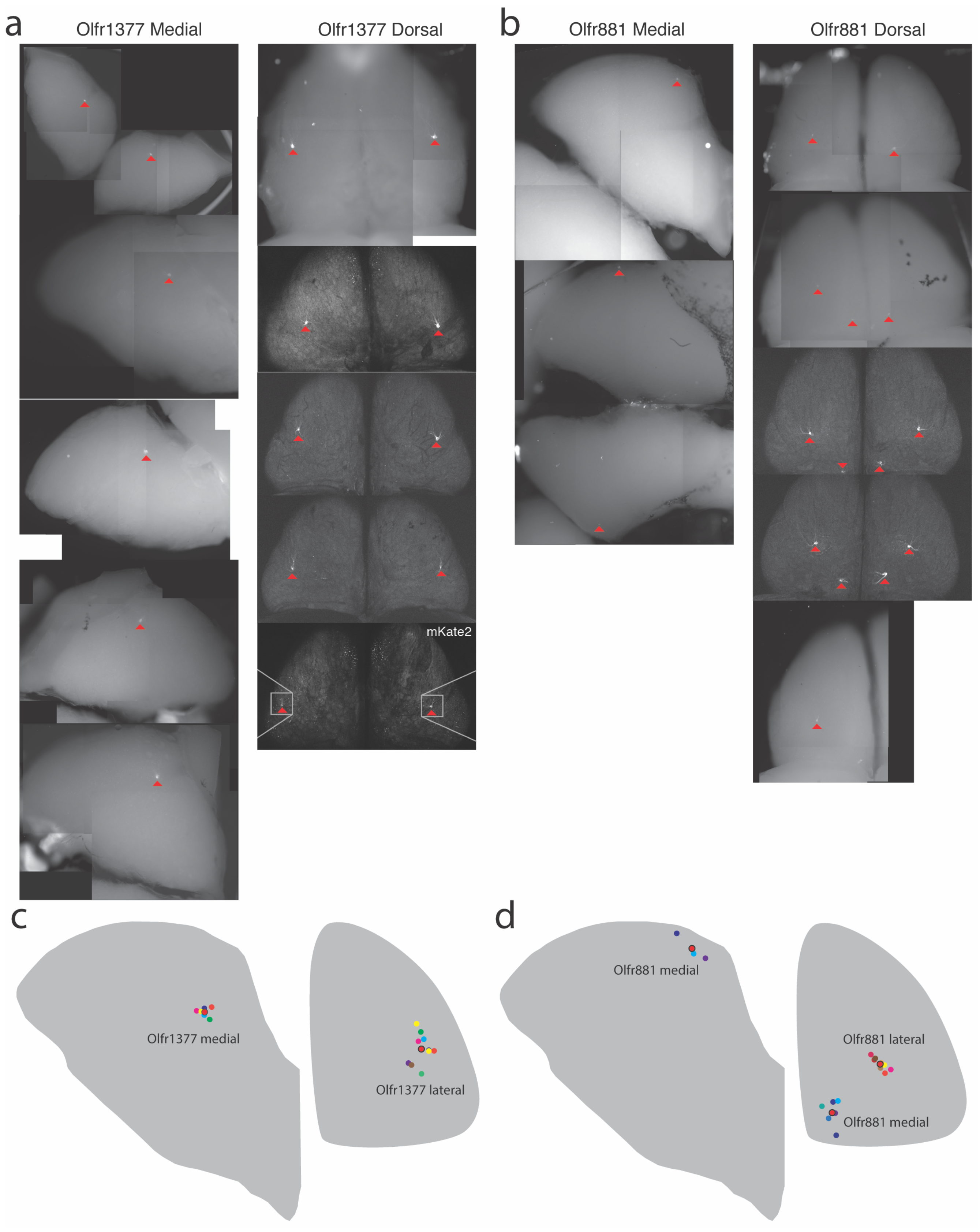
a
Olfr1377 Medial
Olfr1377 Dorsal
mKate2
b
Olfr881 Medial
Olfr881 Dorsal
c
Olfr1377 medial
Olfr1377 lateral
d
Olfr881 medial
Olfr881 lateral
Olfr881 medial

Fig. S14. Position of Olfr1377 and Olfr881 glomeruli. (**a**) Whole-mount stitched epifluorescence images (together with whole-mount confocal maximal intensity projections from fig. S13) reconstructing the medial (n = 7 glomeruli) and dorsal (n = 10 glomeruli) faces of Olfr1377-IRES-mKate2 mice OBs. Red arrows indicate glomerulus. (**b**) Whole-mount stitched epifluorescence images (together with whole-mount confocal maximal intensity projections from fig. S13) reconstructing the medial (n = 9 glomeruli) and dorsal (n = 8 glomeruli) faces of Olfr881-IRES-mKate2 mice OBs. Red arrows indicate glomerulus. (**c**) Positions of Olfr1377 fluorescent glomeruli as recorded from the whole-mount OB images. Red bordered circles indicate the mean position of the individual replicates. (**d**) Same as (c) for Olfr881.

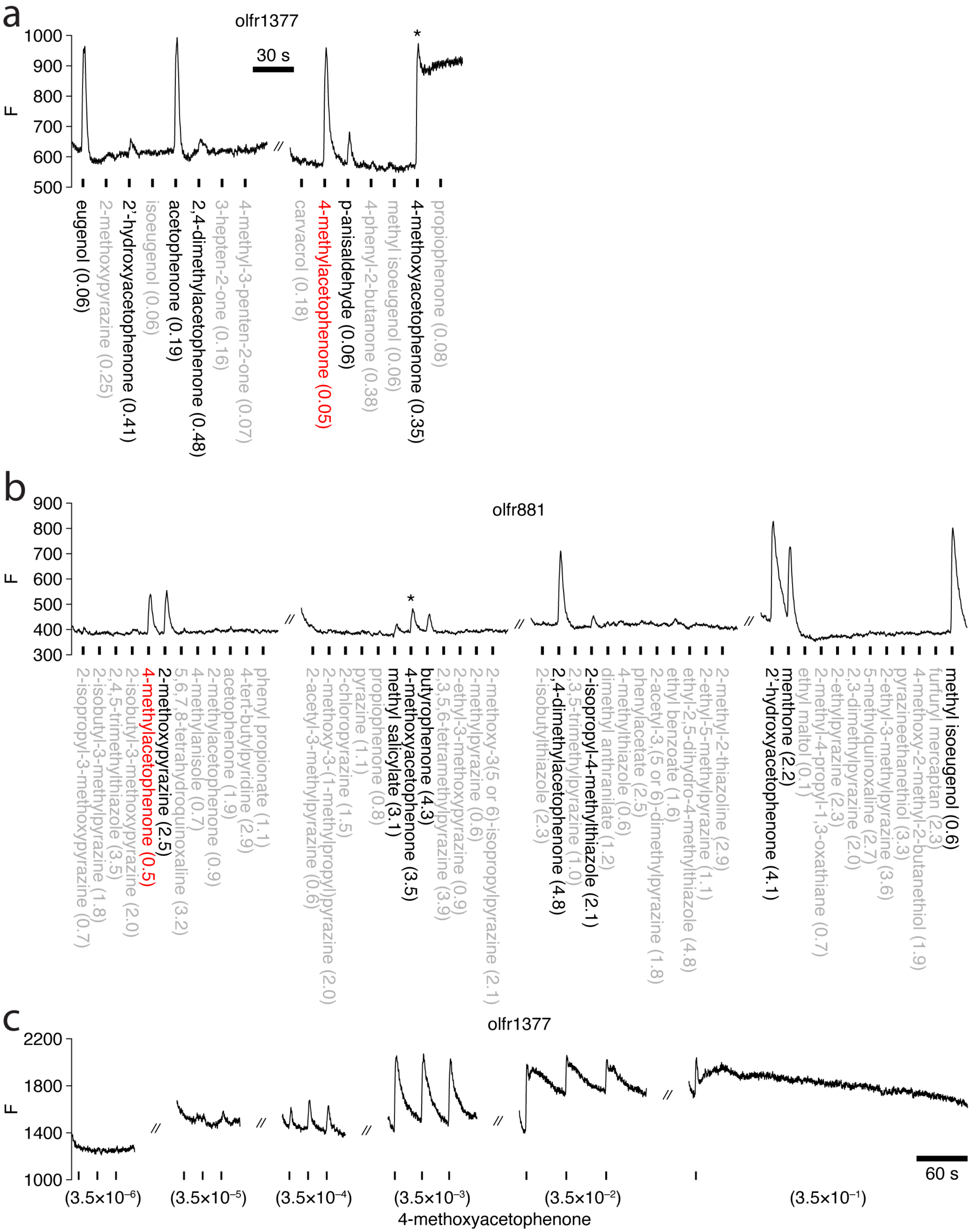
a
olfr1377
1000
900
800
700
600
500
F
30 s
*
//
eugenol (0.06)
2-methoxypyrazine (0.25)
2'-hydroxyacetophenone (0.41)
isoeugenol (0.06)
acetophenone (0.19)
2,4-dimethylacetophenone (0.48)
3-hepten-2-one (0.16)
4-methyl-3-penten-2-one (0.07)
carvacrol (0.18)
4-methylacetophenone (0.05)
p-anisaldehyde (0.06)
4-phenyl-2-butanone (0.38)
methyl isoeugenol (0.06)
4-methoxyacetophenone (0.35)
propiophenone (0.08)
b
olfr881
900
800
700
600
500
400
300
F
*
2-isopropyl-3-methoxypyrazine (0.7)
2-isobutyl-3-methylpyrazine (1.8)
2,4,5-trimethylthiazole (3.5)
2-isobutyl-3-methoxypyrazine (2.0)
4-methylacetophenone (0.5)
2-methoxypyrazine (2.5)
5,6,7,8-tetrahydroquinoxaline (3.2)
4-methylanisole (0.7)
2-methylacetophenone (0.9)
acetophenone (1.9)
4-tert-butylpyridine (2.9)
phenyl propionate (1.1)
2-acetyl-3-methylpyrazine (0.6)
2-methoxy-3-(1-methylpropyl)pyrazine (2.0)
2-chloropyrazine (1.5)
pyrazine (1.1)
propiophenone (0.8)
methyl salicylate (3.1)
4-methoxyacetophenone (3.5)
butyrophenone (4.3)
2,3,5,6-tetramethylpyrazine (3.9)
2-ethyl-3-methoxypyrazine (0.9)
2-methylpyrazine (0.6)
2-methoxy-3(5 or 6)-isopropylpyrazine (2.1)
2-isobutylthiazole (2.3)
2,4-dimethylacetophenone (4.8)
2,3,5-trimethylpyrazine (1.0)
2-isopropyl-4-methylthiazole (2.1)
dimethyl anthranilate (1.2)
4-methylthiazole (0.6)
phenylacetate (2.5)
2-acetyl-3,(5 or 6)-dimethylpyrazine (1.8)
ethyl benzoate (1.6)
ethyl-2,5-dihydro-4-methylthiazole (4.8)
2-ethyl-5-methylpyrazine (1.1)
2-methyl-2-thiazoline (2.9)
2'-hydroxyacetophenone (4.1)
menthone (2.2)
ethyl maltol (0.1)
2-methyl-4-propyl-1,3-oxathiane (0.7)
2-ethylpyrazine (2.3)
2,3-dimethylpyrazine (2.0)
5-methylquinoxaline (2.7)
2-ethyl-3-methylpyrazine (3.6)
pyrazineethanethiol (3.3)
4-methoxy-2-methyl-2-butanethiol (1.9)
furfuryl mercaptan (2.3)
methyl isoeugenol (0.6)
c
olfr1377
2200
1800
1400
1000
F
60 s
(3.5×10⁻⁶)
(3.5×10⁻⁵)
(3.5×10⁻⁴)
(3.5×10⁻³)
(3.5×10⁻²)
(3.5×10⁻¹)
4-methoxyacetophenone

Fig. S15. In vivo two-photon GCaMP6s response kinetics and concentration-dependence of Olfr1377 and Olfr881 glomeruli. (**a**) Raw two-photon GCaMP6s fluorescence of a mKate2-labeled Olfr1377 glomerulus during sequential presentation of odorants in pseudorandom order to a compound heterozygous Olfr1377-IRES-mKate2; OMP-IRES-tTA; tetO-GCaMP6s mouse. * marks long-term activation by 4-methoxyacetophenone. Red text highlights response to 4-methylacetophenone. Odorants presented at an estimated concentration on the order of 10^{-1} nM. (**b**) Similar to (a) for a mKate2-labeled Olfr881 glomerulus imaged in a compound heterozygous Olfr881-IRES-mKate2; OMP-IRES-tTA; tetO-GCaMP6s mouse. Note the lack of long-term activation by 4-methoxyacetophenone. Odorants presented at an estimated concentration on the order of 10^{0} nM. (**c**) Raw two-photon GCaMP6s fluorescence of a mKate2-labeled Olfr1377 glomerulus during sequential presentation of increasing concentrations of 4-methoxyacetophenone, revealing long-term activation with 3.5×10^{-2} nM and higher concentrations.
