# Supplementary material for: Decoding the olfactory map through targeted transcriptomics links murine olfactory receptors to glomeruli
Source: Nat Commun. 2022 Sep 1;13:5137. doi: 10.1038/s41467-022-32267-3 (PMC9437035; doi:10.1038/s41467-022-32267-3)
Supplement: Supplementary file 3 — Reporting Summary [file 41467_2022_32267_MOESM3_ESM.pdf]

nature portfolio

Corresponding author(s): Kevin Zhu, Matt Wachowiak, Hiroaki Matsunami

Last updated by author(s): May 5, 2022

Reporting Summary

Nature Portfolio wishes to improve the reproducibility of the work that we publish. This form provides structure for consistency and transparency in reporting. For further information on Nature Portfolio policies, see our Editorial Policies and the Editorial Policy Checklist.

Statistics

For all statistical analyses, confirm that the following items are present in the figure legend, table legend, main text, or Methods section.

n/a	Confirmed	
☐	☒	The exact sample size (*n*) for each experimental group/condition, given as a discrete number and unit of measurement
☐	☒	A statement on whether measurements were taken from distinct samples or whether the same sample was measured repeatedly
☐	☒	The statistical test(s) used AND whether they are one- or two-sided<br>*Only common tests should be described solely by name; describe more complex techniques in the Methods section.*
☐	☒	A description of all covariates tested
☐	☒	A description of any assumptions or corrections, such as tests of normality and adjustment for multiple comparisons
☐	☒	A full description of the statistical parameters including central tendency (e.g. means) or other basic estimates (e.g. regression coefficient) AND variation (e.g. standard deviation) or associated estimates of uncertainty (e.g. confidence intervals)
☐	☒	For null hypothesis testing, the test statistic (e.g. *F*, *t*, *r*) with confidence intervals, effect sizes, degrees of freedom and *P* value noted<br>*Give P values as exact values whenever suitable.*
☐	☒	For Bayesian analysis, information on the choice of priors and Markov chain Monte Carlo settings
☒	☐	For hierarchical and complex designs, identification of the appropriate level for tests and full reporting of outcomes
☐	☒	Estimates of effect sizes (e.g. Cohen's *d*, Pearson's *r*), indicating how they were calculated

Our web collection on statistics for biologists contains articles on many of the points above.

Software and code

Policy information about availability of computer code

Data collection: STAR and RSEM were used to process raw sequencing reads

Data analysis: Key code for the project has been made publicly available at https://github.com/kanazian/obmap

R (v4.1.1)
RStudio (v1.4.1717)
R packages: tidyverse (1.3.1), plotly (4.9.4.1), uwot (0.1.10), reshape2 (1.4.4), gtools (3.9.2), dunn.test (1.3.5), Biostrings (2.60.2), seqinr (4.2-8), MCMCpack (1.5-0), Rcpp (1.0.7), edgeR (3.34.1), e1071 (1.7-9), geomorph (4.0.1), raster(3.4-13), doParallel (1.0.16), patchwork (1.1.1), SC3 (1.20.0), SingleCellExperiment (1.14.1), scater (1.20.1), xgboost(1.5.0.2)
Snakemake (v3.5.5)
STAR (v2.7.0d)
RSEM (v1.3.1)
Blender (v2.83)
Jalview (v2.11.1.4)
Modeller (v9.25)
GraphPad Prism (v9.0.0)
MATLAB 2018b (v9.5)
Adobe Photoshop (v20.0.6)

For manuscripts utilizing custom algorithms or software that are central to the research but not yet described in published literature, software must be made available to editors and reviewers. We strongly encourage code deposition in a community repository (e.g. GitHub). See the Nature Portfolio guidelines for submitting code & software for further information.

Data

Policy information about availability of data

All manuscripts must include a data availability statement. This statement should provide the following information, where applicable:

- Accession codes, unique identifiers, or web links for publicly available datasets
- A description of any restrictions on data availability
- For clinical datasets or third party data, please ensure that the statement adheres to our policy

All sequencing data will be made publicly available under NCBI BioProject PRJNA773191 upon publication. Additional raw and processed data that support the findings of this study are available from the corresponding authors upon request.

Field-specific reporting

Please select the one below that is the best fit for your research. If you are not sure, read the appropriate sections before making your selection.

☒ Life sciences ☐ Behavioural & social sciences ☐ Ecological, evolutionary & environmental sciences

For a reference copy of the document with all sections, see nature.com/documents/nr-reporting-summary-flat.pdf

Life sciences study design

All studies must disclose on these points even when the disclosure is negative.

Sample size	No statistical methods were used to predetermine sample size. The sample sizes in this study reflect the number required for replication while remaining within time and budget constraints.
Data exclusions	The set of genes analyzed in this study did not include target genes which were: 1. not sufficiently enriched by the targeted enrichment process, as indicated by a mean value in all spatial samples below 1TPM, 2. genes which did not display a significant difference against a uniform distribution, 3. genes which were manually deemed as having exceptionally high expression, potentially indicative of ectopic expression in non-target cell types.
Replication	All replications of the method and data are displayed in this paper with n=3 animals for dorsoventral and mediolateral sections and n=6 for anteroposterior sections were successful.
Randomization	Samples were randomized during processing to limit systemic batch effects.
Blinding	Data collection and analysis were not performed blind to the conditions of the experiments because experiments and analysis were performed by the same person with no specific expected outcomes.

Reporting for specific materials, systems and methods

We require information from authors about some types of materials, experimental systems and methods used in many studies. Here, indicate whether each material, system or method listed is relevant to your study. If you are not sure if a list item applies to your research, read the appropriate section before selecting a response.

Materials & experimental systems

n/a	Involved in the study	
☐	☒	Antibodies
☐	☒	Eukaryotic cell lines
☒	☐	Palaeontology and archaeology
☐	☒	Animals and other organisms
☒	☐	Human research participants
☒	☐	Clinical data
☒	☐	Dual use research of concern

Methods

n/a	Involved in the study	
☒	☐	ChIP-seq
☒	☐	Flow cytometry
☒	☐	MRI-based neuroimaging

Antibodies

Antibodies used	Cell Signaling #5364
Validation	Previously tested and published from our lab. Cell Signaling document certifies product has met quality control standards and approved for immunofluorescence.

Eukaryotic cell lines

Policy information about cell lines

Cell line source(s)	The Matsunami lab was previously responsible for the generation of the Hana3A cell line
Authentication	The cell line was validated by STR testing
Mycoplasma contamination	Cell lines tested negative for mycoplasma contamination.
Commonly misidentified lines (See ICLAC register)	No commonly misidentified cell liens were used in this study.

Animals and other organisms

Policy information about studies involving animals; ARRIVE guidelines recommended for reporting animal research

Laboratory animals	Mus musculus: C57BL6 (p20-22), Olfr1377-IRES-mKate2 (p21-180), Olfr881-IRES-mKate2 (p21-180), OMP-IRES-tTA(p21-180), tetO-GCaMP6s (p21-180) mice were used in this study. Both male and female mice were used.
Wild animals	This study did not involve the use of wild animals
Field-collected samples	This study did not involve the use of field-collected sample
Ethics oversight	The Duke University Institutional Animal Care and Use Committee and the University of Utah provided ethical approval and guidance for all animal handling and tissue collection procedures performed in this study

Note that full information on the approval of the study protocol must also be provided in the manuscript.
