# Supplementary material for: Decoding the olfactory map through targeted transcriptomics links murine olfactory receptors to glomeruli
Source: Nat Commun. 2022 Sep 1;13:5137. doi: 10.1038/s41467-022-32267-3 (PMC9437035; doi:10.1038/s41467-022-32267-3)
Supplement: Supplementary file 2 — Peer Review File [file 41467_2022_32267_MOESM2_ESM.pdf]

nature portfolio

Peer Review File

Decoding the olfactory map through targeted transcriptomics links murine olfactory receptors to glomeruli

Open Access This file is licensed under a Creative Commons Attribution 4.0 International License, which permits use, sharing, adaptation, distribution and reproduction in any medium or format, as long as you give appropriate credit to the original author(s) and the source, provide a link to the Creative Commons license, and indicate if changes were made. In the cases where the authors are anonymous, such as is the case for the reports of anonymous peer reviewers, author attribution should be to 'Anonymous Referee' followed by a clear attribution to the source work. The images or other third party material in this file are included in the article's Creative Commons license, unless indicated otherwise in a credit line to the material. If material is not included in the article's Creative Commons license and your intended use is not permitted by statutory regulation or exceeds the permitted use, you will need to obtain permission directly from the copyright holder. To view a copy of this license, visit http://creativecommons.org/licenses/by/4.0/.

Editorial Note: This manuscript has been previously reviewed at another journal that is not operating a transparent peer review scheme. This document only contains reviewer comments and rebuttal letters for versions considered at *Nature Communications* . Mentions of the other journal have been redacted.

REVIEWERS' COMMENTS

Reviewer #2 (Remarks to the Author):

The authors have further clarified their findings and edited the text accordingly to address the concerns raised. In my opinion, the revised manuscript is ready for publication.
Dinu Albeanu

Reviewer #3 (Remarks to the Author):

I was enthusiastic in supporting the publication of this paper in [redacted]. The authors appeared to have modified the paper to address some of the questions I raised in that review. I am enthusiastic in supporting the publication of this paper in Nature Communications.
